# Simultaneous miRNA and mRNA transcriptome profiling of human myoblasts reveals a novel set of myogenic differentiation-associated miRNAs and their target genes

**DOI:** 10.1186/1471-2164-14-265

**Published:** 2013-04-18

**Authors:** Petr Dmitriev, Ana Barat, Anna Polesskaya, Mary J O’Connell, Thomas Robert, Philippe Dessen, Thomas A Walsh, Vladimir Lazar, Ahmed Turki, Gilles Carnac, Dalila Laoudj-Chenivesse, Marc Lipinski, Yegor S Vassetzky

**Affiliations:** 1 UMR 8126, Univ. Paris-Sud 11, CNRS, Institut de Cancérologie Gustave-Roussy, 39, rue Camille-Desmoulins, Villejuif 94805, France; 2 INSERM U-1046, 371 Avenue du Doyen Gaston Giraud, Montpellier, F-34295, France; 3 Institut de Cancérologie Gustave-Roussy, Villejuif, F-94805, France; 4 Bioinformatics and Molecular Evolution Group, School of Biotechnology, Dublin City University, Glasnevin, Dublin 9, Ireland; 5 CNRS FRE 3377, CEA Saclay, Gif-sur-Yvette, F-91191, France

**Keywords:** microRNA, Skeletal muscle, Myogenesis, Myoblast

## Abstract

**Background:**

miRNA profiling performed in myogenic cells and biopsies from skeletal muscles has previously identified miRNAs involved in myogenesis.

**Results:**

Here, we have performed miRNA transcriptome profiling in human affinity-purified CD56+ myoblasts induced to differentiate *in vitro*. In total, we have identified 60 miRNAs differentially expressed during myogenic differentiation. Many were not known for being differentially expressed during myogenic differentiation. Of these, 14 (miR-23b, miR-28, miR-98, miR-103, miR-107, miR-193a, miR-210, miR-324-5p, miR-324-3p, miR-331, miR-374, miR-432, miR-502, and miR-660) were upregulated and 6 (miR-31, miR-451, miR-452, miR-565, miR-594 and miR-659) were downregulated. mRNA transcriptome profiling performed in parallel resulted in identification of 6,616 genes differentially expressed during myogenic differentiation.

**Conclusions:**

This simultaneous miRNA/mRNA transcriptome profiling allowed us to predict with high accuracy target genes of myogenesis-related microRNAs and to deduce their functions.

## Background

MicroRNAs (miRNAs) are 20–25 nt-long non-coding RNAs transcribed predominantly by the RNA polymerase II [1,2]. miRNAs play an important role in regulating gene expression at the protein and mRNA levels. They have been previously shown to inhibit translation initiation and elongation and induce deadenylation and degradation of mRNA (for review see [3]). In addition, there are indications that miRNAs may also activate translation [4].

Along with transcription factors, miRNAs contribute to skeletal myogenesis (for review see [5]). Myogenic microRNAs miR-1, miR-133a/b and miR-206 (also called MyomiRs, as suggested by [6]) regulate myogenic differentiation and proliferation of myogenic cells by targeting important regulators of myogenesis [7,8] (for review see [6,9]). MiR-1 and miR-133a are expressed in both skeletal and cardiac muscles [7,10], while miR-133b and miR-206 are expressed solely in skeletal muscles [10].

Most miRNAs expressed in skeletal muscles are found to be upregulated during myogenesis. These include miR-24 [11], miR-26a [12], miR-27b [13], miR-29b/c [14], miR-143 [15], miR-181 [16], miR-208b/499 [17], miR-214 [18], miR-322/424 and miR-503 [19], miR-486 [20], miR-682 [21]. Only few known miRNAs are involved in the regulation of myogenic differentiation are downregulated during myogenesis. These include miR-125b [22] and miR-221/222 [23] (for review see [5,24,25]).

Altogether, myogenesis-related miRNAs have been studied during myogenic differentiation of human primary myoblasts [14,15,26,27] and in various model systems, e.g. the immortalized murine cell line C2C12 [12,16,20,28], murine primary satellite cells and myogenic progenitor cells [21] as well as quail myoblasts [29]. Several myogenesis-related miRNAs have been discovered or confirmed by expression profiling of developing muscular tissues in humans [30], pigs [31] and in the common carp [32] (Table 1).

**Table 1:** microRNAs (MR-miRs) found to be up- and downregulated during myogenic differentiation in this study

	**microRNA**	**This study**	**Human (homo sapiens)**	**Mouse (Mus musculus)**	**Pig**	**Common carp**
1	*miR-1*	↑↑↑	enriched in sk. muscle[15] ↑pMyo diff. [26] ↑ muscle development [30]	↑ C2C12 diff [7,8,10,33] ↑ pMyo diff [33]	↑ muscle development [31]	↑ muscle development [32]
2	*miR-15a*	↑↑	(this study)	↓ pMyo diff [33]	↑ muscle development [31]	
3	*miR-16*	↓	(this study)	↓ C2C12 diff [33] ↓ pMyo diff [33]	↑ muscle development [31]	
4	*miR-21*	↑	↑ pMyo diff. [26]	-		↑ muscle development [32]
5	*miR-22*	↑	(this study)	↑ C2C12 diff [33] ↑ pMyo diff [33]	↑ muscle development [31]	
6	**miR-23b (n)**	↑	(this study)			
7	*miR-24*	↑	↑pMyo diff. [26]	↑pMyo diff, ↑ C2C12 diff [11,33] ↑ C2C12 diff [28,33]		enriched in heart [32]
8	*miR-26a*	↑	↑pMyo diff. [26]	↑ C2C12 diff [12,28] ↑ pMyo diff [33]		↑ muscle development [32]
9	*miR-27a*	↑	↑pMyo diff. [26]	↑ pMyo diff [33]	↑ muscle development [31]	↓ muscle development [32]
10	*miR-27b*	↑	(this study)	↑ pMyo [13,33] ↑ C2C12 diff [28,33]		
11	**miR-28 (n)**	↑	(this study)	-		
12	*miR-29a*	↓	(this study)	↓ C2C12 diff [28] ↑ C2C12 diff [33]		
13	*miR-30a-5p*	↑↑	(this study)	↑ C2C12 diff [28,33]		
14	miR-30a-3p	↑	(this study)	↑ C2C12 diff [33]		
15	*miR-30c*	↑	↑pMyo diff. [26]	↑ C2C12 diff [33]	↑ muscle development [31]	
16	miR-30d	↑↑	(this study)	↑ pMyo diff [33]		
17	miR-30e-3p	↑	(this study)	↑ pMyo diff [33]		
18	**miR-31 (n)**	↓	(this study)			
19	**miR-98 (n)**	↑	(this study)			
20	miR-99a	↓	(this study)	↑ C2C12 diff [33] ↑ pMyo diff [33]		
21	*miR-101*	↑↑	(this study)	-	↑ muscle development [31]	
22	**miR-103 (n)**	↑	(this study)			
23	**miR-107 (n)**	↑	(this study)	-		
24	miR-126	↑↑	(this study)	↓ C2C12 diff [33]		
25	*miR-128b*	↑↑↑	↑ pMyo diff. [27]	↑ C2C12 diff [28] ↑ pMyo diff [33]		
26	*miR-133a*	↑↑↑	enriched in sk. muscle[15]↑pMyo diff. [26] ↑ muscle development [30]	↑ C2C12 diff [8,10,33] ↑ pMyo diff [33]	↑ muscle development [31]	↑ muscle development [32]
27	*miR-133b*	↑↑↑	enriched in sk. muscle[15] ↑ muscle development [30]	↑ C2C12 diff [8,10] ↑ pMyo diff [33]		
28	miR-140	↑	(this study)	↑ pMyo diff [33]		
29	*miR-152*	↑	(this study)	↑ C2C12 diff [28]		
30	*miR-155*	↓↓	(this study)	↓ C2C12 diff [34,35] ↓ pMyo diff [33]		
31	*miR-181b*	↑	(this study)	↑ C2C12 diff [16,28,33] ↑ pMyo diff [33]		enriched in the heart [32]
32	miR-183	↓	(this study)	↓ C2C12 diff [33]		
33	miR-192	↑↑	(this study)	↑ pMyo diff [33]		
34	**miR-193a (n)**	↑↑	(this study)	-		
35	miR-204	↓↓	↓ pMyo diff. [27]	-		
36	*miR-206*	↑↑	enriched in sk. muscle[15] ↑ muscle development [30]	↑ C2C12 diff [8,10,33] ↑ pMyo diff [33]		↑ muscle development
37	**miR-210 (n)**	↑	(this study)			
38	*miR-221*	↓	↑pMyo diff. [26]↓ pMyo diff. [27]	↓quail myoblasts diff, ↓ C2C12 diff [29]		
39	*miR-222*	↓	↑pMyo diff. [26] ↓ pMyo diff. [27]	↓quail myoblasts diff, ↓ C2C12 diff [29] ↓ C2C12 diff [28]		↓ muscle development [32]
40	miR-320	↓	(this study)	↑ pMyo diff [33]		
41	**miR-324-3p (n)**	↑↑	(this study)			
42	**miR-324-5p (n)**	↑	(this study)			
43	**miR-331 (n)**	↑	(this study)	-		
44	miR-339	↓	(this study)	↑ C2C12 diff [33] ↑ pMyo diff [33]		
45	miR-361	↑	(this study)	↑ pMyo diff [33]		
46	*miR-362*	↑↑	(this study)	↑ C2C12 diff [28]		
47	**miR-374 (n)**	↑	(this study)			
48	**miR-432 (n)**	↑	(this study)			
49	**miR-451 (n)**	↓↓↓	(this study)			
50	**miR-452 (n)**	↓↓	(this study)			
51	*miR-500*	↑↑	(this study)	↑ C2C12 diff [28,33] ↑ pMyo diff [33]		
52	*miR-501*	↑↑↑	(this study)	↑ C2C12 diff [28,33]		
53	**miR-502 (n)**	↑↑	(this study)	-		
54	*miR-503*	↑	(this study)	↑ C2C12 diff [19,28,33] ↑ pMyo diff [33]		
55	*miR-532*	↑↑	(this study)	↑ C2C12 diff [28,33] ↑ pMyo diff [33]		
56	**miR-550**	↓	↓ pMyo diff. [27]	-		
57	**miR-565 (n)**	↓	(this study)	-		
58	**miR-594 (n)**	↓	(this study)			
59	**miR-659 (n)**	↓↓↓	(this study)	-		
60	**miR-660 (n)**	↑↑↑	(this study)	-		

In **bold **are human-specific MR-miRs (hMR-miRs, 16 in total); *Italicized* are microRNAs shown to be differentially expressed during myogenesis in non- human cells; microRNAs with a known function in myogenesis are underscored. (n): microRNAs that we have shown to be differentially expressed during myogenesis for the first time in our study. The number of arrows corresponds to the level of up- or downregulation of microRNAs. One arrow: fold change 1 to 3 fold, two arrows: fold change 3 to 7 times, three arrows: fold change 7 to infinity.

To perform transcriptome profiling of myogenic cells, it appears interesting to use primary myogenic cells cultivated *in vitro* rather than whole muscle tissues. This approach minimizes biases caused by the presence of inflammatory, connective tissue-derived and other non-myogenic cells. Nevertheless, totally avoiding contamination by non-myogenic cells is difficult unless clonal populations of immortalized myogenic cells are used. An alternative to generating immortalized lines of myogenic cells is the utilization of primary cell population enriched for myogenic precursors. However, in previous miRNA and mRNA expression profiling reports, primary skeletal myoblasts cultivated *in vitro* have not been enriched for myogenic cells prior to analysis.

In the present study, we have thus decided to profile miRNA expression in cultures of CD56+ primary myoblasts and myotubes isolated from healthy individuals using an affinity purification procedure [35]. A total of 60 miRNAs were found to be differentially expressed during myogenic differentiation induced by serum starvation of which 20 had not been previously associated with myogenesis. In addition, we have performed microarray mRNA transcriptome profiling on the same myoblasts and myotubes samples as used for miRNA expression profiling in order to assign targets for these miRNAs.

## Results

### Identification of new myogenesis-related MIRNA (MRMIRNA) in human primary myoblasts

**Figure 1:**
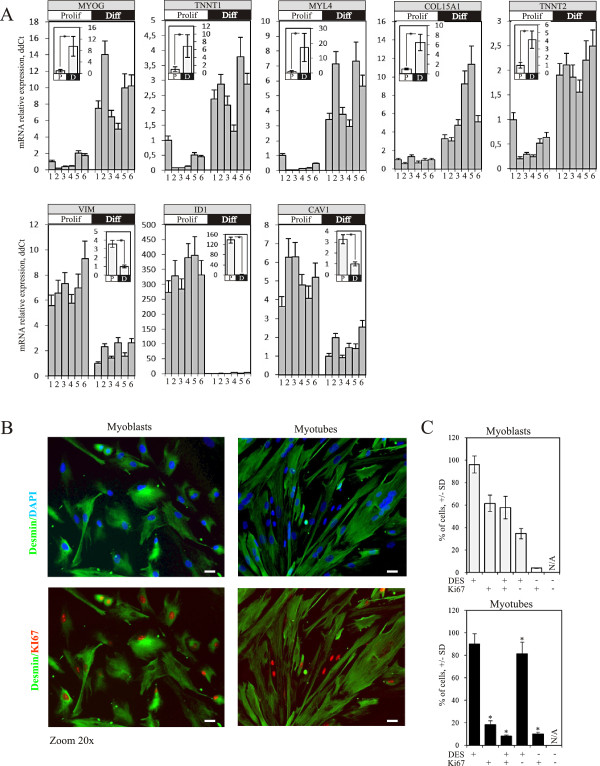
**Expression of myogenic differentiation markers and miRNA profiling. A**. Expression of muscle differentiation markers MYOG (myogenin, myogenic factor 4, p-value=0.0016), TNNT1 (Troponin T type 1, skeletal slow, p-value=0.00064), MYL4 (myosine light chain 4, p-value=0.0016), COL15A1 (collagen, type XV, alpha 1, p-value=0.013), TNNT2 (troponin T type 2 (cardiac), p-value=4.8x10^-6), VIM (vimentin, p-value=0.00034), ID1 (inhibitor of DNA binding 1, p-value=1.9x10^-5) and CAV1 (caveolin 1, p-value=0.00015) in proliferating myoblasts (labeled as P) and differentiated myotubes (labeled as D) was measured using qRT-PCR, normalization was performed using ΔΔCt method using GAPDH as a control gene and proliferating sample #1 as a reference sample (expression level 1). The average of three independent experiments is shown. Numbers from 1 to 6 indicate the sample number (for full description refer to Table S1). Insets indicate the average of 6 samples of proliferating myoblasts (P) and differentiated myotubes (D). Asterisk corresponds to p-values<0.05. Error bars correspond to standard deviation (SD) in the case of individual samples and standard error of the mean (SEM) in the case of average expression levels (inset). **B**. Results of immunofluorescence microscopy analysis of cells stained with anti-Ki67 (red), and anti-Desmin (green) antibodies and DAPI nuclear staining (blue) showing normal cellular localization of these proteins in proliferating myoblasts and differentiated myotubes, 20x magnification. Bar=10 μm. **C**. The number of Ki67+ cells is significantly reduced during myogenic differentiation *in vitro*. Results of quantification of the DES+ and Ki67+ cells representing 300 individual cells. Statistically significant difference between cell cultures of proliferating myoblasts and differentiated myotubes is indicated by an asterisk (t-test p-value<0.05).

To identify new myogenesis-related miRNAs in human cells, CD56+ myogenic precursors were isolated from skeletal muscle biopsies of six healthy individuals (Additional file 1). These were then induced to differentiate *in vitro* using serum starvation and the progression of myogenic differentiation was monitored by following the expression of Myogenin (MYOG) and other myogenic differentiation markers including ID1, TNNT1, TNNT2, MYL4, COL15A1, VIM and CAV1 (Figure 1A). The disappearance of proliferating cells and concomitant emergence of myotubes was monitored using Ki67 staining and microscopic observation (Figure 1B and C).

We observed a statistically significant difference of the expression levels of myogenesis-related markers in proliferating or differentiated myogenic cells isolated from different healthy subjects. This difference was considered as a natural heterogeneity of gene expression levels in different subjects that might be explained by their age, sex, physical activity or diet preferences, therefore, all samples analyzed were included in the study.

**Figure 2:**
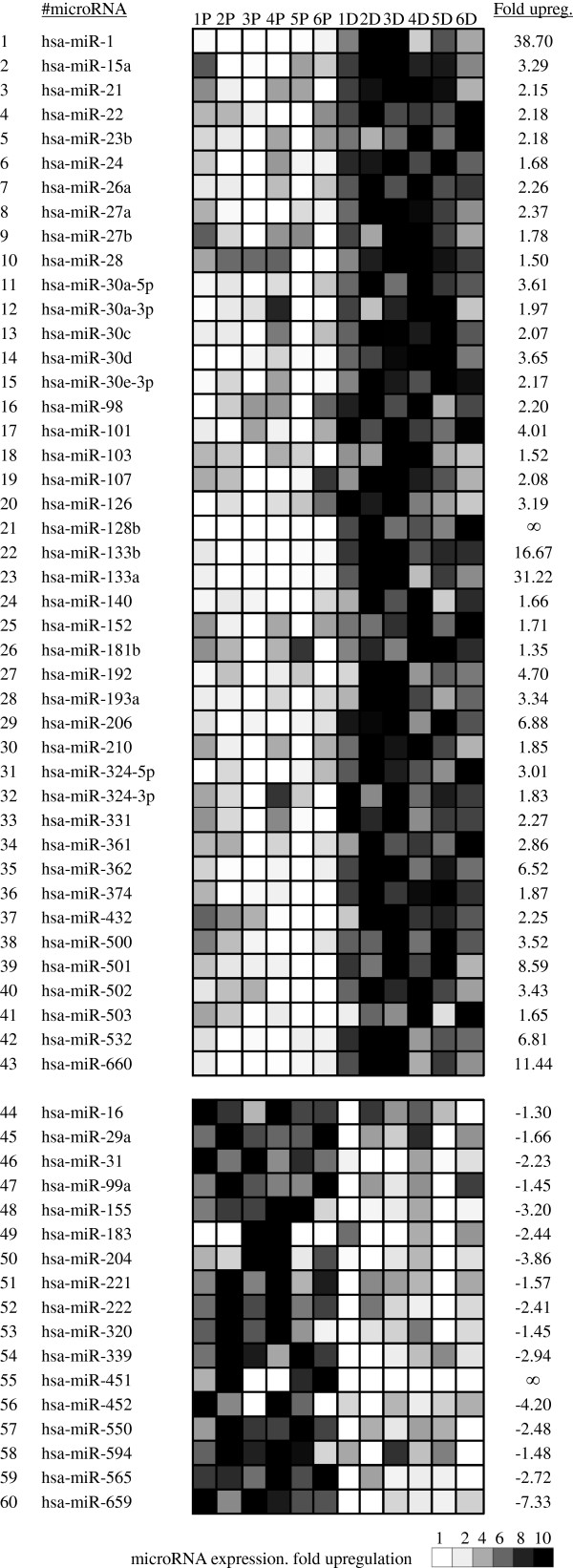
**miRNA profiling in proliferating myoblasts (left) and differentiated myotubes (right). **Out of 365 miRNAs tested, this table shows 60 miRNAs that were differentially expressed in myotubes as compared to myoblasts. Gray levels indicates the expression level of microRNA in each individual sample tested. Labels 1P to 6P correspond to samples of proliferating myoblasts described in Table S1, labels 1D to 6D correspond to the samples of differentiated myotubes described in Additional file 1: Table S1.

The expression of 365 different microRNAs was then profiled in human primary myoblasts and myotubes using TaqMan Low Density Array (TLDA) (Additional file 2). In total, 60 miRNAs were found to be differentially expressed during myogenic differentiation with p-values ranging from 3.4x10^-5^ to 5x10^-2^ (Figure 2). These will be referred to as myogenesis-related miRNAs (MR-miRs). Sequences and corresponding TaqMan probe codes for these microRNAs can be found in the Additional file 3.

The majority (43 out of 60) of MR-miRs were found to be upregulated of which 14 had not been previously reported as involved in the regulation of myogenesis either in humans or in other species (Table 1). Of the 17 MR-miRs that were found to be downregulated during myogenesis, 6 had not been previously associated with myogenic differentiation.

### MRNA transcriptome profiling and functional classification of differentially expressed genes

Until now, mRNA expression profiling of human myoblasts has been performed exclusively on primary cells [36]. However, primary skeletal myoblasts have not been enriched for myogenic cells prior to analysis. To avoid analyzing mixed populations of myoblasts and non-myogenic cells, we have used RNA extracted from CD56+ myoblasts purified from muscular tissues. Then, using Agilent transcriptome 44 K microarrays, we identified 6,616 differentially expressed genes of which 3,445 and 3,245 were down- or up-regulated, respectively (Additional file 4). In most cases, a functional class could be assigned to these transcripts using the DAVID Gene Ontology database (Additional file 5) [37,38].

In agreement with previous reports, the majority of upregulated genes were found to control metabolism, myogenesis, insulin receptor signaling or to positively regulate transcription (Table 2). Genes involved in the control of cell cycle, DNA damage response, angiogenesis, cell motility and invasion as well as protein modification and NF-kB signaling belonged to the group of genes downregulated during myogenic differentiation (Table 2). Genes involved in the regulation of ubiquitination/proteolysis, protein transport/localization, transcription regulation and apoptosis were found to be either up- or down-regulated (Table 3). The complete list of genes found to be differentially expressed during in vitro myogenic differentiation of human primary myoblasts can be found in the Additional file 4.

**Table 2:** Specific functional classes of genes differentially expressed during myogenic differentiation

	**Specific functional classes**	**Unique genes/cluster**	**% targets**	**P-value**	**FDR**	**Cluster code**
	**Upregulated**					
**1**	**Myogenesis**					
	*- muscle contraction*	81	8,64	3,881E-19	7,23E-16	1_up
	*- muscle development*	145	13,79	6,37E-09	1,19E-05	2_up
	*- embryonic development*	85	11,76	0,0001374	0,255618	3_up
**2**	**Metabolism**					
	*- cellular respiration*	179	12,85	2,21E-24	4,12E-21	4_up
	*- phosphate metabolism*	217	13,82	2,459E-16	4,11E-13	5_up
	*- cofactor biosynthesis*	57	12,28	5,177E-09	9,64E-06	6_up
	*- carbohydrate metabolism*	50	18,00	1,556E-06	0,002898	7_up
**3**	**Signaling**					
	*- insulin receptor signaling*	132	19,70	2,61E-07	0,000486	8_up
**4**	**Transcription**					
	*- transcription (+) reg*	220	15,00	2,624E-07	0,000489	9_up
	**Downregulated**					
**1**	**cell cycle**					
	*- cell cycle regulation*	285	35,09	1,04E-51	1,95E-48	1_down
	*- chromosome segregation*	42	26,19	2,51E-15	4,77E-12	2_down
	*- M-phase*	31	32,26	3,75E-07	0,000702	3_down
	*- interphase*	34	20,59	2,9E-08	5,43E-05	4_down
	*- DNA replication*	38	34,21	8,04E-06	0,015052	5_down
	*- centrosome cycle*	19	15,79	1,6E-06	0,002987	6_down
**2**	**DNA damage response**					
	*- DNA damage response*	253	39,53	2,8E-38	5,23E-35	7_down
	*- telomere maintenance*	24	29,17	3,02E-05	0,056435	8_down
**3**	**Wound healing/angiogensis**					
	*- wound healing*	58	44,83	5,52E-08	0,000103	9_down
	*- angiogenesis*	54	55,56	1,14E-05	0,021416	10_down
**4**	**Cell motility/migration**					
	*- cell motility*	87	44,83	1,72E-05	0,032183	11_down
**5**	**Protein modification/assembly**					
	*- protein glycosylation*	36	36,11	0,001102	2,041321	12_down
	*- protein modification*	88	46,59	4,82E-05	0,090071	13_down
**6**	**Metabolism**					
	*- nucleotide biosynthesis*	84	41,67	5,39E-07	0,001009	14_down
**7**	**Signaling**					
	*- response to calcium*	40	40,00	2,91E-05	0,054402	15_down
	*- NF-kB*	65	50,77	0,003016	5,494941	16_down
	*- phosphorylation*	163	38,65	3E-07	0,000561	17_down
**8**	**Transcription**					
	*- RNA splicing*	135	36,30	3,48E-17	6,51E-14	18_down

Subclasses are italicized, the cutoff value for differentially expressed genes was set to 1.2 fold change.

**Table 3:** Common functional classes unifying genes up- and downregulated during myogenic differentiation

	**Common functional classes**	**Unique genes/cluster**	**UP/DOWN**	**% miRNA targets**	**P-value**	**FDR**	
**1**	**Protein modification/assembly**						
	*ubiquitination/proteolysis*	305	**UP**	16,07	4,118E-23	7,67E-20	10*_up*
		162	DOWN	39,51	2,97E-05	0,055652	19_down
	*protein complex assembly*	131	**UP**	9,16	0,0001135	0,21125	11*_up*
		177	DOWN	30,51	7,01E-17	2,11E-13	20_down
**2**	**Transport/localization**						
	*transport/localization*	243	**UP**	16,05	1,299E-17	2,42E-14	12*_up*
		279	DOWN	41,22	1,27E-11	2,39E-08	21_down
**3**	**Transcription**						
	*transcription regulation*	458	**UP**	13,32	7,64E-10	1,42E-06	13*_up*
		36	DOWN	41,67	0,000311	0,57965	22_down
	*transcription (-) reg,*	148	**UP**	11,49	9,994E-07	0,001862	14*_up*
		203	DOWN	36,45	1,19E-09	2,23E-06	23_down
	*chromatin organization/modification*	86	**UP**	9,30	0,0005777	1,070598	15*_up*
		153	DOWN	30,72	5,88E-23	1,1E-19	24_down
**4**	**Apoptosis**						
	*apoptosis*	216	**UP**	17,59	8,615E-07	0,001605	16*_up*
		216	DOWN	38,43	1,26E-08	2,36E-05	25_down
**5**	**Signaling**						
	*reg, of kinase activity*	204	**UP**	15,69	1,362E-06	0,002537	17*_up*
		233	DOWN	40,77	2,22E-06	0,00416	26_down
**6**	**Cell morphogenesis**						
	*cytoskeleton organization*	130	**UP**	14,62	0,0002361	0,438978	18, 19*_up*
		193	DOWN	35,23	1,05E-17	1,96E-14	27_down

Subclasses are italicized, the cutoff value for differentially expressed genes was set to 1.2 fold change.

### Predication of myogenesis-related MIRNA target genes

A common practice to boost target gene predictions accuracy is to use several prediction algorithms and then to combine their predictions. However, this approach has been proved inefficient [39]. Therefore, we have used a single algorithm, e.g. RNA22 [40] to predict miRNA targets. RNA22 has been chosen for its low rate of false-positive predictions as compared to other algorithms [39]. For each miRNA, the number of target predictions varied between 261 and 3,058.

**Figure 3:**
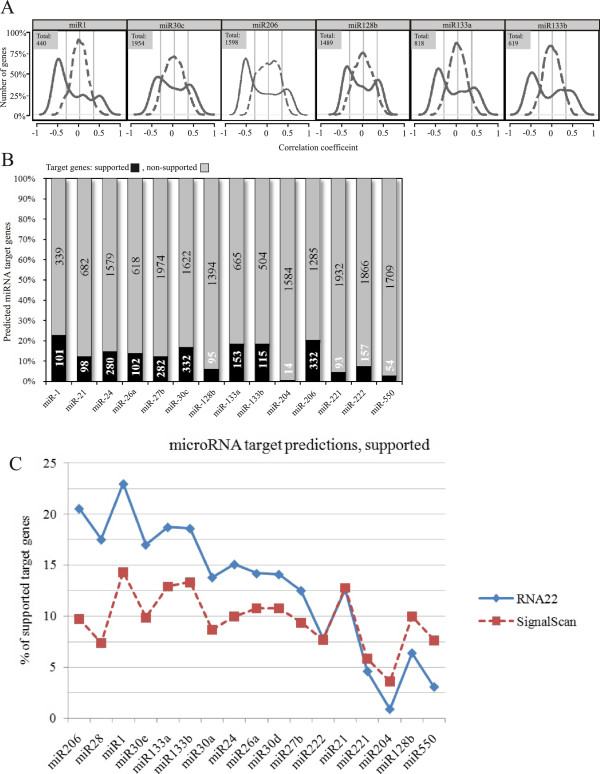
**Bioinformatic predictions of microRNA target genes. A**. Density plot of Pearson correlation coefficients between expression of miRNA and their target genes (continuous line) predicted by RNA22 algorithm. Number of genes is expressed as% of total predictions indicated within each plot. The distribution of Pearson correlations is clearly shifted towards negative values; the density plot of Pearson correlation coefficient after permutation of the list of microRNAs (dashed line). In this case the distribution is centered around zero. Several examples are shown, for other microRNAs see the Additional file 6. **B.** Diagram showing the proportion of bioinformatic predictions made by RNA22 algorithm (taken for 100%) that were supported by transcriptome profiling (black). Light grey shows unsupported predictions. The graphs corresponding to TargetScan predictions are presented in the Additional file 8. **C.** Comparison of the accuracy of target gene predictions by RNA22 and TargetScan algorithms.% of target genes predicted by both algorithms and supported by transcriptome data is shown.

According to previous reports, the proportion of correct predictions can be as low as 10% [41,42] (for recent review see [43]. To increase the accuracy of bioinformatic predictions, we used a simultaneous mRNA/miRNA expression profiling approach described in [42,44,45]. Based on the fact that MiRNA regulate gene expression by inhibiting translation or inducing deadenylation of mRNAs followed by their degradation (for review see [46,47]), we reasoned that the expression levels of mRNA and miRNA should be inversely correlated if one regulates the other. Indeed, for upregulated MR-miRNA, the majority of their predicted target genes was found to be underexpressed. Similarly, most downregulated MR-miRNAs had upregulated predicted targets. Indeed, for every microRNA tested, the distribution of Pearson correlation coefficients of its predicted target genes was shifted towards negative values. This was the case neither for randomly selected genes, nor when the sample correspondence was permuted (Figure 3A and the Additional file 6) which was used to establish a significance threshold of Pearson correlation coefficients. Target genes with correlation coefficients below the threshold had less than 5% chances to be random. We call these genes “supported targets” to underscore the fact that the prediction was supported by mRNA transcriptome profiling (Additional file 7). On average, 11% of microRNA target genes predicted by the RNA22 algorithm were “supported” by mRNA transcriptome profiling (Figure 3B).

We then compared the accuracy of predictions made by RNA22 and TargetScan, another popular miRNA target prediction algorithm. As an indication of accuracy, we have used a percentage of predicted targets that were supported by transcriptome data. For 11 of 17 target genes, RNA22 demonstrated higher percentage of predictions supported by transcriptome data, for 2 more microRNAs the difference in the accuracy of predictions was insignificant (Figure 3C and Additional file 8).

### QRT-PCR validation of supported MIRNA target genes

**Figure 4:**
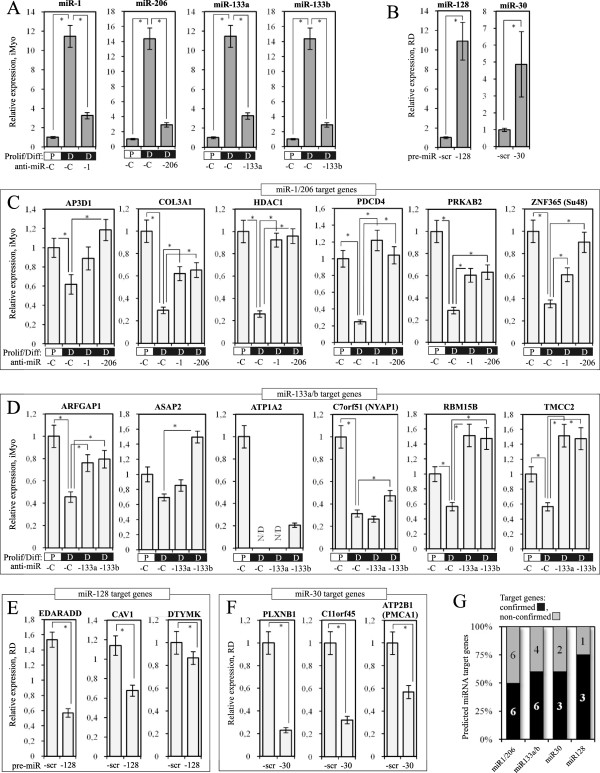
**Confirmation of bioinformatic predictions of miR targets. **Human immortalized myoblasts (iMyo) cultivated in growth medium (P) or differentiation medium for 3 days (D) were transfected with either control LNA (anti-miR-C) or LNA against miR-1 and miR-206, miR-133a and miR-133b (**A**). Human rhabdomyosarcoma cells (RD) were transfected with plasmids coding for miR-128 and miR-30 precursors or scrambled sequence (scr) (**B**). Then the expression of corresponding microRNA and their randomly selected target genes was tested using qRT-PCR, normalization was performed using ΔΔCt method using GAPDH as a control gene and proliferating sample #1 as a reference sample (expression level 1). Transcriptome-supported target genes of miR-1/206 (**C**) and miR-133a/b (**D**) were downregulated during normal myogenic differentiation but not when it was accompanied by the transfection with corresponding anti-miRs. Transcriptome-supported target genes of miR-128 and miR-30 were downregulated when human rhabdomyosarcoma cells were transfected with lentiviral constructs overexpressing miR-128 (**E**) and miR-30 (**F**). The average of three independent experiments is shown. (*) indicates p-value <0.05; (**G**): Diagram showing the proportion of supported predictions that were qRT-PCR validated in this study. Black: validated targets, gray: non-validated targets.

To demonstrate the relevance of our miRNA target predictions, we altered the expression of several microRNAs and measured the expression of their predicted target genes, supported by the transcriptome data. To illustrate the impact of miRNA repression on the expression of their target genes, we have chosen to inhibit the expression of four well-characterized myogenic microRNAs miR-1, -133a, -133b and -206 in human immortalized myoblasts using LNA (Locked Nucleic Acids) antisense microRNAs (anti-miRs). Human immortalized myoblasts were differentiated *in vitro* and then transfected separately with anti-miRs targeting miR-1, miR-133a, miR-133b and miR-206. miR-1 and miR-206 have the same seed sequence and, therefore, target very similar lists of genes. The same is true for miR-133a and miR-133b. We have then randomly selected 12 and 10 genes predicted to be targeted by miR-1/206 and miR-133a/b respectively and supported by our transcriptome data and tested their expression using qRT-PCR in the cells transfected with corresponding anti-miRs. Repressing miR-1/206 resulted in the upregulation of six of its targets: AP3D1 (adaptor-related proteins complex 3, delta 1 subunit), COL3A1 (collagen, type III, alpha 1), HDAC1 (histone deacetylase 1), PDCD4 (programmed cell death 4), PRKAB2 (protein kinase, AMP-activated beta 2 non-catalytic subunit) and ZNF365 (zinc finger protein 365). These target genes were considered as novel “qRT-PCR validated” target genes of miR-1 and -206 (Figures 4A). Six other target genes supported by our transcriptome data including ATP2B1, C11orf45, FOXP, G2E3, MBOAT7 and TUBB could not be validated via qRT-PCR experimentally, as we did not observe significant changes of their expression following anti-miR transfection (data not shown).

In the case of miR-133a and -133b, immortalized myotube transfection anti-miRs resulted in the upregulation of six of ten randomly selected target genes, supported by our transcriptome data. These novel qRT-PCR validated targets of miR-133a and -133b include ASAP2 (ArfGAP with SH3 domain, ankyrin repeat and PH domain 2), ARFGAP1 (ADP-ribosylation factor GTPase activating protein 1), ATP1A2 (ATPase, Na+/K+ transporting, alpha 2 polypeptide), C7orf51 (chromosome 7 open reading frame 51), RBM15B (RNA binding motif protein 15B) and TMCC2 (transmembrane and coiled-coil domain family 2) (Figures 4B). Four other target genes supported by our transcriptome data including LMCD1, NAT9, PRPF38A and SYNGR2 could not be validated via qRT-PCR, as we did not observed significant changes of their expression following anti-miR transfection (data not shown).

To illustrate the impact of miRNA overexpression on the expression of their target genes, we have selected two microRNAs, miR-30 and -128, that were previously shown to be upregulated during myogenic differentiation [26,27], but their function in myogenesis is not well-established. Two plasmids coding for the corresponding pre-miRNAs or scrambled sequence were transfected separately into human rhabdomyosarcoma cells, and the expression of their target genes was then tested via qRT-PCR. Ectopic overexpression of miR-128 led to the downregulation of three of its four target genes, randomly selected among the genes supported by our transcriptome data. These novel qRT-PCR validated miR-128 target genes include EDARADD (EDAR-associated death domain), CAV1 (Caveolin 1) and DTYMK (deoxythymidylate kinase) (Figures 4C). One transcriptome-supported miR-128 target gene, RBM15B, could not be validated using qRT-PCR, as we did not observed significant changes in its expression level following miRNA precursor tranfection (data not shown).

Similarly, overexpressing miR-30 led to the downregulation of three of its five randomly selected transcriptome-supported targets. These qRT-PCR validated miR-30 targets include PLXNB (plexin B2), C11orf45 (chromosome 11 open reading frame 45) and ATP2B1 (ATPase, Ca++ transporting, plasma membrane 1) (Figures 4D). Two remaining miR-30 target genes, PPIA and CASD1 could not be validated via qRT-PCR, as we did not observed significant changes in its expression level following miRNA precursor transfection (data not shown).

Overall, at least 50% of the supported target genes tested in this study appeared to be regulated by their corresponding microRNA either directly or indirectly which is significantly better than the result of ordinary bioinformatic prediction with only 10% of correctly predicted target genes.

### Functional analysis of MR-MIRNA target genes supported by transcrptome data

After functional classification of genes differentially expressed during myogenic differentiation *in vitro*, we thought to determine the proportion of microRNA-controlled genes within each functional class. Almost 30% of all protein coding genes contain miRNA seeds in their 3^′^UTRs and are, therefore, potential miRNA targets [48]. Indeed, we have found that a significant proportion of genes differentially expressed during myogenic differentiation appeared to be controlled by microRNAs. However, the proportion of genes targeted by microRNAs was not the same within different functional classes. For example, 50% to 55% of genes related to angiogenesis and NF-kB signaling were potentially targeted by miRNAs, while in the case of myogenesis, potential miRNA targets represented only 8.6% of genes (Table 2). Interestingly, more miRNA targets were found among downregulated than upregulated genes. For example, among apoptosis-related genes, 17.6% of upregulated and 38.4% of downregulated genes were found to be targeted by miRNAs (Table 3). This observation prompted us to search for known functions of each supported target gene of every MR-miRNA described in this study using DAVID functional annotation tool [37,38].

#### miR-1 and miR-206

**Figure 5:**
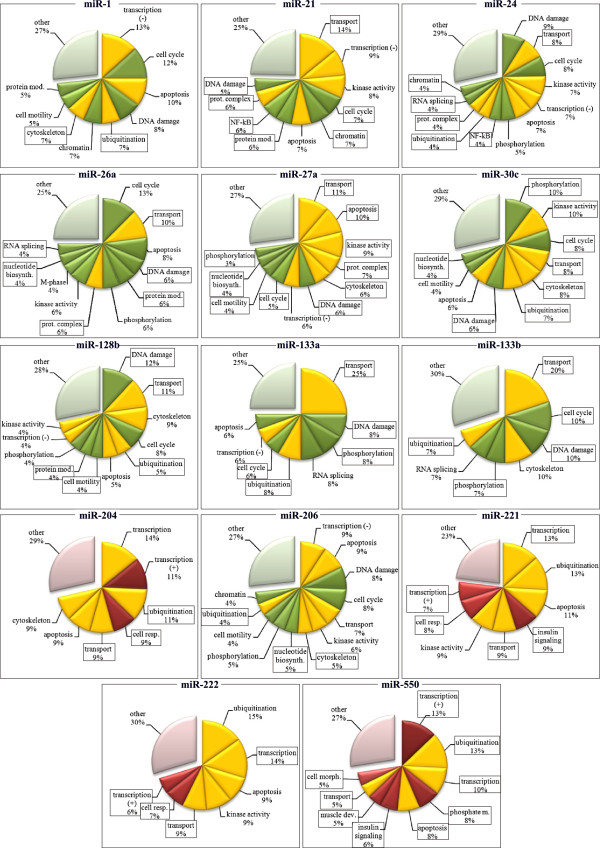
**Predicted functions of MR-miRs. **Green: functions downregulated during myogenic differentiation, Red: functions upregulated during myogenic differentiation. Yellow: functions that are both up- and downregulated during myogenic differentiation. Framed are the functions that have not been previously ascribed to a given microRNA.

It has been previously demonstrated that miR-1 and -206 target genes linked to the regulation of chromatin modifications [8], transcription [20,49] and cell cycle [50]. Functional analysis of predicted miR-1 target genes supported by transcriptome data indicated that these microRNAs might also target genes involved in the regulation of apoptosis, DNA damage response, cell motility and protein modification, cell signaling and kinase activity [33]. Our own functional analysis of predicted target genes of miR-1 and -206 supported by transcriptome analysis confirmed that miR-1 and miR-206 might target genes involved in in these biological processes. In addition, miRNA-1 and -206 might target genes implicated in cytoskeleton organization, ubiquitination and nucleotide biosynthesis (Figure 5, Additional file 9).

#### miR-21

It has been demonstrated that miR-21 targets genes involved in in apoptosis [51,52] and regulation of kinase activity [53-56] and regulation of gene expression [57]. Here we confirm that miR-21 might target genes involved in these biological processes and might also target genes involved in transport, cell cycle regulation, chromatin assembly, protein modification, NF-kB signaling, protein complex assembly and DNA damage (Figure 5, Additional file 9).

#### miR-24

It has been demonstrated that miR-24 is involved in DNA damage response [58,59], regulation of cell cycle progression [59,60], regulation of kinase activity and protein phosphorylation [61], transcription [62] and apoptosis [63]. We confirm that miR-24 might control genes implicated in these biological processes. In addition, miR-24 might control genes implicated in the regulation of transport , NF-kB signaling, protein ubiquitination and complex assembly, RNA splicing and chromatin modification (Figure 5, Additional file 9).

#### miR-26a

It has been previously demonstrated that miR-26a target genes are implicated in cell cycle control [64], apoptosis [65], regulation of kinase activity and protein phosphorylation [66], as well as chromatin modification [12]. Here we confirm that miR-26a might target genes involved in the regulation of cell cycle, apoptosis, kinase activity and protein phosphorylation, but not in chromatin modification. In addition, miR-26a might target genes involved in the regulation of transport, DNA damage response, protein modification and protein complex assembly, as well as nucleotide biosynthesis and RNA splicing (Figure 5, Additional file 9).

#### miR-27a

miR-27b is involved in the regulation of transcription by inhibiting the expression of various transcription factors [13,67-70]. Here we confirm the role of miR-27b as an important transcriptional regulator, in addition, our predictions suggest that miR-27a might be involved in the regulation of intracellular transport, apoptosis, kinase activity, protein complex assembly, cytoskeleton organization and other biological functions including DNA damage response, cell cycle, motility, nucleotide biosynthesis and protein phosphorylation (Figure 5, Additional file 9).

#### miR-30c

miR-30 was shown to induce apoptosis [71] and regulate cell motility by influencing extracellular the matrix remodelling process [72-74]. This microRNA is also known to regulate protein modification e.g. sumoylation [75] and transcription [72,73,76,77]. We here confirm that miR-30c might target genes participate in the regulation of apoptosis and cell motility regulation but not transcription control. Instead, our predictions suggest that miR-30 might target genes involved in the regulation of protein phosphorylation and kinase activity, cell cycle control, intracellular transport, cytoskeleton organization, protein ubiquitination, DNA damage response and nucleotide biosynthesis (Figure 5, Additional file 9).

#### miR-128b

It has been previously demonstrated that miR-128b targets genes involved in the regulation of cell cycle [78,79], transcription [78,79] and apoptosis [79,80]. In addition, transcriptome analysis of cell transfected with miR-128 revealed an alteration of the expression of genes implicated in cytoskeleton organization, kinase activity and protein phosphorylation [81]. Here we confirm the target genes of miR-128 might be involved in these biological functions. In addition, our predictions suggest that miR-128 might target genes in DNA damage response, transport, protein modification and ubiquitination and cell motility (Figure 5, Additional file 9).

#### miR-133a and miR-133b

Previously, these microRNAs have been shown to control genes involved in the regulation of transcription [8,82], cytoskeleton structure [83-87], apoptosis [88] and mRNA splicing [89]. Here we confirm that miR-133a/b might target genes involved in these biological functions. In addition, miR-133a/b might target genes involved in intracellular transport, cell cycle regulation, DNA damage response, protein phosphorylation and ubiquitination (Figure 5, Additional file 9).

#### miR-204

It has been previously shown that miR-204 is involved in the regulation of transcription [90-92], controls cell migration cytoskeleton organization [92-94]. miR-204 has been also shown to be involved in regulation of apoptosis by targeting multiple genes [95] and in the regulation of cell signaling [94], [96]. Here we confirm that miR-204 might target genes involved in transcription regulation, cytoskeleton organization and apoptosis but not cell signaling and cell migration. Furthermore, our predictions suggest that this microRNA might target genes involved in the control of ubiquitination, cellular respiration and intracellular transport (Figure 5, Additional file 9).

#### miR-221 and miR-222

Previously, these microRNAs have been show to target genes involved in the process of protein degradation [97], apoptosis [98], kinase activity [99,100], cell cycle progression [29] and cell motility [101]. We here confirm that miR-221 and -222 might target genes involved in protein degradation (via ubiquitination), apoptosis, and kinase activity regulation, but not cell cycle progression or cell motility. In addition, miR-221 and -222 might also target genes that play a role in the regulation of transcription, insulin signaling, intracellular transport and cellular respiration (Figure 5, Additional file 9).

#### miR-550

No target genes and functions of this microRNA were not previously described. Our predictions suggest that miR-550 might target genes involved in the regulation of transcription, protein ubiquitination, phosphate metabolism, apoptosis, insulin signaling, muscle development, intracellular transport and cellular morphology (Figure 5, Additional file 9).

The full list of supported target genes of MR-miRs with and without assigned functions can be found in the Additional file 7.

## Discussion and conclusion

MicroRNAs, particularly miR-1, -206 and -133a/b, play crucial roles in myogenesis [6]. These are called MyomiRs to underscore their importance in myogenesis (for review see [24]). Previously, high-throughput profiling of miRNA expression performed in human myoblasts have resulted in identification of miRNAs differentially expressed during myogenic differentiation [26,27]. Besides that, a larger number of miRNAs have been found to be differentially expressed during myogenic differentiation of mouse C2C12 myoblasts [19,28], mouse myogenic progenitors [18,21] or at various stages of development of skeletal muscles in pig [31] or in the common carp [32].

Here, we have compared miRNA expression profiles in human proliferating primary CD56+ myoblasts and differentiated myotubes. Sixty differentially expressed miRNAs were identified (MR-miRs). Twenty of these had not been previously implicated in myogenesis. Conversely, several miRNAs previously reported as related to myogenic differentiation were not identified in the present study. These included miR-214 [18], hsa-miR-424 (identical to mmu-miR-322) [19], miR-29b/c [14], miR-143, miR-208a [15], miR-208b, miR-499 [17], miR-125b [102] (Additional file 10). One miRNA, miR-682, previously shown to be upregulated in mouse myogenic precursors [21] could not be tested here. The difficulties to confirm previously reported data for several miRNAs associated with myogenesis in model organisms could be due to species-specific characteristics in the myogenesis program or could originate from differences in experimental conditions. Indeed, high-throughput miRNA expression profiling has been previously performed using mixed cell populations isolated from skeletal myoblasts [26,27]. The resulting presence of non-myogenic cells, including fibroblasts and inflammatory cells, could have altered the resulting miRNA expression profiles. It thus appears that the existing repertoire of myogenic miRNAs is still incomplete or contaminated with miRNAs unrelated to myogenesis. For example, we have demonstrated that miR-221 and miR-222 were downregulated during myogenic differentiation of human myoblasts while others reported their upregulation [26]. Our results are in agreement with previous reports demonstrating that these microRNAs are downregulated during myogenesis in mice [28,29].

In addition to microRNA expression profiling, we have profiled mRNA expression in myogenic cells in the course of myogenic differentiation. Transcriptome analysis of myogenesis has been previously reported by others. In mice, several models have been used for transcriptome profiling. These include C2C12 immortalized cell line considered as the “golden standard” of *in vitro* myogenic differentiation [103-107], primary mouse myoblasts [108] and an *in vivo* model of muscle regeneration following injury [109]. In humans, the only published mRNA expression profiling has been performed on primary cells grown *in vitro*[36]. The transcriptome profiling of human myoblasts in the course of myogenic differentiation *in vitro* described here, has two important advantages over the previously published study. Firstly, we used CD56+ myogenic cells that allowed us to exclude from the analysis genes unrelated to myogenesis. Secondly, our study is the first example of a simultaneous miRNA/mRNA expression profiling of myogenic cells.

Simultaneous miRNA/mRNA was used here to identify novel target genes of myogenesis-related microRNAs. This approach has been proven efficient for increasing the precision of bioinformatics predictions for miRNA targets [42], and it has been applied previously to identify miRNA target genes in neural tissue [45] and cancer cell lines [44,110], but not in myogenic cells or muscle tissue.

Several methods are used to validate the target genes of miRNA. These include qRT-PCR, luciferase assays and western blot (for review see [111] and [112]). Luciferase assay based on a reporter plasmid containing miRNA recognition sites in the 3^′^ UTR of the luciferase gene is the method used to demonstrate the direct inhibition of mRNA expression by miRNA. However this method does not provide information as to whether the miRNA-dependent regulation occurs at the level of transcript stability or at the level of translation, nor the direct miRNA:mRNA interaction demonstrated by this method does guarantee the physiological significance of the observed miRNA effect. Here, to validate the predicted target genes, we have used qRT-PCR validation approach. This method is more physiological than luciferase assay as it measures the expression of target gene from their natural genomic context, although it can not discriminate between direct and indirect effects of miRNA expression. Hereby, we have randomly selected 32 genes targeted by 6 different microRNAs including miR-1, -206, -133a, -133b, -128 and -30 and tested their expression using pRT-PCR in the cells where corresponding microRNA was ectopically overexpressed or inhibited. We have demonstrated the miRNA-dependent inhibition for 18 of these target genes. We called these genes “qRT-PCR validated” to formally distinguish them from simply “validated”, as the latter term is usually reserved for miRNA target genes validated using luciferase reporters.

On average, we estimate that the lists of predicted target genes supported by transcriptome data contain at least 50% of genes that will change their expression, according to our predictions, in response to the alterations of microRNA expression.

Using the lists of target genes supported by transcriptome data we have deduced functional impact of MR-miRs using DAVID functional annotation tool [37,38]. Our predictions generally corresponded to already known functions of MR-miRs demonstrated by others, but also allowed us to make suggestions about novel functions of MR-miRs. It has to be noted, that these predictions have to be handled with caution for two reasons: (i) according to our estimation, among target genes supported by transcriptome analysis, up to 50% might not react to miRNA alteration, as predicted; (ii) the functions of MR-miR have been deduced using bioinformatic prediction algorithms that can not substitute for a true experimental validation of microRNA functions.

To summarize, we have discovered 20 human miRNAs not previously known to be differentially expressed during myogenic differentiation and confirmed 40 myogenesis-related microRNAs. We then used an approach that included a simultaneous miRNA/mRNA expression profiling to predict targets genes of these microRNAs with more than 50% accuracy, although whether these are direct or indirect targets of miRNA remains unknown. Finally, we deduced novel functions of these microRNA from the known functions of their target genes. Further studies are necessary to confirm our predictions of functional contribution of newly discovered miRNAs to the process of myogenic differentiation.

## Methods

### Cell culture conditions and transfection

Primary human myoblasts were isolated from skeletal muscles of healthy subjects as described in [35], for details see Additional file 4: Table S4), purified with an immuno-magnetic sorting system (Miltenyi Biotec, USA) using an anti-CD56/NCAM antibody according to the manufacturer’s instructions. CD56-positive myoblasts were seeded in collagen-coated Petri dishes (P1) and cultured in DMEM, 10% FCS, 1% Ultroser G, at 37°C with 5% CO_2_.All experiments were carried out between P1 and P5 to avoid cell senescence. Myoblast purity was determined by staining for Desmin. Purified myoblasts were plated in collagen-coated Petri dishes and cultured in growth medium containing DMEM supplemented with 20% fetal bovine serum at 37°C in humidified atmosphere with 5% CO_2_. Myogenic differentiation of confluent cells was induced after 5 days by changing to DMEM containing 2% FBS (differentiation medium). Cells were kept in differentiation medium for 3 days. Myogenic fusion index at day three was 37-70%. Human immortalized myoblasts (iMyo) (kind gift of Dr. V. Mouly) were grown and differentiated as described in [113]. RD and TE671 (a kind gift of Dr. S. Leibowitz) were grown as described [114]. phrGFP-1 vector was from Stratagen, pcDNA3.2/V5 hsa-mir-128 (#26308) [115] and pCMV-miR30 (#20875) [116] vectors were provided by Addgene. Transient transfection of RD cells was performed in 6-well plate format using Lipofectamine 2000 according to manufacturer’s instructions with minor modification: 600.000 cells were added to the transfection mixture prepared directly in the cell culture plate. To transfect human immortallized myoblasts, anti-sense LNA inhibitors of miR-1, -206, 133a and b, and the irrelevant control were purchased from Exiqon (Denmark) and were mixed with LipoRNAiMAX (Invitrogen) in 6-well plates at 100nM final concentration, using 500 000 cells per well.

### Reverse transcription and real-time PCR

For microRNA expression analysis 400 ng of total RNA purified via Trizol (Invitrogen) was converted into cDNA using 8 independent pools of primers (#4384791, Applied Biosystems, AB) and TaqMan microRNA Reverse transcription kit (#4366596, AB). cDNA was quantified using via qPCR using TaqMan 2x Universal PCR Master Mix, No AmpErase UNG (#4324018, AB) and human microRNA panel version 1.0 TLDA (TaqMan Low Density Array, AB), data were acquired on AB7900HT Real-Time PCR machine. Sequences of microRNAs and corresponding codes of TaqMan assays can be found in Additional file 2. For mRNA expression analysis total RNA was isolated from 2x10^6^ proliferating myoblasts, or differentiated myotubes using Trizol (Invitrogen) and reverse transcribed using the High Capacity cDNA Archive kit (Applied Biosystems, AB) according to the manufacturer protocol. cDNA was mixed with 2x Taqman PCR mix (AB) and amplified customized TLDA (TaqMan Low Density Array) using Abiprism 7900HT apparatus (AB). In case of miRNA and mRNA the expression level was calculated from Ct values using ΔΔCt method using GAPDH as a control [117]. The calculation of SEM in case of average of multiple samples has been done as described in [118].

### Transcriptome profiling and data processing

Human primary myoblasts were sacrificed directly on plates at 30% confluency using Trizol, RNA was prepared using organic extraction and ethanol precipitation as described [119] followed by silica column cleanup on silica columns (Nucleospin RNA Extraction kit, Macherey Nagel). RNA extracted from individual myoblast lines was Cy5-labeled, mixed with with a pool of RNA samples labeled with Cy3 and hybridized to Gene Expression microarrays (4x44k #G4112F Agilent) and scanned as instructed by the manufacturer. Scanned images were then analysed using the Feature Extraction Software (Agilent), the subsequent gene expression data treatment was performed using R and Bioconductor. Spots with intensity lower than 50 or lower than background in more than 50% of biological replicates have been removed from further analysis. The background correction and intensity normalization procedures were applied for the remaining ~30 000 probes using the Bioconductor package vsn [120]. A background offset and a scaling factor for each array and dye channel were calculated using the least squares regression procedure, then the generalized log-transformation was applied. The ordinary least squares regression approach is based on the assumption that “most genes are not differentially expressed”. However, in the case of myogenic differentiation, where many genes are differentially expressed, this assumption does not hold. Therefore, to apply the above mentioned approach to myogenic differentiation, the vsn least squares model has first been applied to a subset of features, and then extended for the whole set of features. To select the subset of features, a pool of samples prepared from five different proliferating myoblast lines before and after myogenic differentiation were hybridized to two additional microarrays. Then 14.358 features that did not exceed the cutoff value of 1.23 fold change between the pools have been selected. To determine the differentially expressed genes, a t-test analysis was conducted using the limma package from Bioconductor [120]. Using this package, a linear model is fitted to the expression data for each gene. An empirical Bayes moderation of the standard errors were performed. This method borrows information across genes in order to arrive at more stable estimates of each individual gene’s variance, even for experiments with small number of arrays. The microarray data related to this paper have been submitted to the Array Express data repository at the European Bioinformatics Institute (http://www.ebi.ac.uk/arrayexpress) under the accession number E-MTAB-1577 (release date 2014-3-22).

### miRNA targets predictions

RNA22 [40] and TargetScan algorithms [121] have been used to predict miRNA target genes in this study. miRNA targets identified by RNA22 have been downloaded from the RNA22 website http://cbcsrv.watson.ibm.com/rna22.html. Predictions were processed in the form of *3*^′^UTR binding sites occurrence matrices, containing the number of 3^′^UTR binding sites for each combination of miRNA (columns) and mRNA (rows).

### Pearson correlation between mRNA and miRNA expression profiles and permutation tests for significance

All the following has been performed using the R software. Pearson correlation values were computed using expression levels of each single transcript available and for each miRNA, found to be differentially expressed during myogenic differentiation in this study. Pearson correlation values were retained for further analysis, if they were corresponding to miRNA and mRNA pares having at least one occurrence in at least one of the two 3^′^UTR binding sites occurrence matrices. To distinguish between biologically significant and random correlations, we performed a permutation test. We performed nine different permutations by calculating correlation values using a miRNA table with permuted sample designations. Then, the distribution of the real and permuted data were graphically plotted. For each miRNA, the 5% percentile of the Pearson correlation obtained with the permuted data was used as a threshold (*th)* for selecting biologically significant target genes. For each differentially expressed miRNA, the Pearson correlation value is compared to *th* and if smaller, the gene is retained as a biologically significant target gene regulated by the miRNA, with a 5% risk of error.

## Competing interests

The authors declare that they have no competing interests.

## Authors’ contributions

YSV and PDm designed the experiments, PDm, AP, TR, TAW, VL, AT, GC and DL-C performed the experiments, PDm, AB, MO’C and PDe conducted bioinformatic analysis of the data, YSV, PDm and ML wrote the manuscript. All authors read and approved the final manuscript.

## Supplementary Material

Additional file 1: Table S1 Samples of myogenic cells used in the study were isolated from six healthy subjects. Click here for file

Additional file 2: Table S2 Results of miRNA expression analysis using TaqMan low density arrays. Click here for file

Additional file 3: Table S3 Sequences of mature microRNAs and corresponding TaqMan probes used in this study. Click here for file

Additional file 4: Table S4 mRNAs differentially expressed during myogenic differentiation of human primary myoblasts. Click here for file

Additional file 5: Table S5 Functional classification of genes differentially expressed during myogenic differentiation. Click here for file

Additional file 6: Figure S1 A. Density plot of Pearson correlation coefficients between expression of miRNA and their target genes (continuous line) predicted by RNA22 algorithm and the the density plot of Pearson correlation coefficient after permutation of the list of microRNAs (dashed line). **B**. Schematic representation of ectopic overexpression/knockdown of miRNA in the cells that served to confirm their targets genes supported by transcriptome data. Click here for file

Additional file 7: Table S6 Target genes of microRNAs differentially expressed during myogenic differentiation, supported by transcriptome analysis of mRNA expression. Click here for file

Additional file 8: Figure S2A Density plot of Pearson correlation coefficients between expression of miRNA and their target genes (blue) predicted by TargetScan algorithm and the density plot of Pearson correlation coefficient after permutation of the list of microRNAs (red). **B**. Diagram showing the proportion of bioinformatic predictions made by TargetScan algorithm (taken for 100%) that were supported by transcriptome profiling (black). Blue bars show unsupported predictions. Click here for file

Additional file 9: Table S8 Functions of miRNA target genes supported by transcriptome data. Functional classification has been performed using DAVID functional annotation tool. Click here for file

Additional file 10: Table S7 microRNAs that were previously shown to be differentially expressed during myogenesis but could not be confirmed in the present study. Click here for file
