# Supplementary material for: Simultaneous miRNA and mRNA transcriptome profiling of human myoblasts reveals a novel set of myogenic differentiation-associated miRNAs and their target genes
Source: BMC Genomics. 2013 Apr 18;14:265. doi: 10.1186/1471-2164-14-265 (PMC3639941; doi:10.1186/1471-2164-14-265)
Supplement: Additional file 1: Table S1 — Samples of myogenic cells used in the study were isolated from six healthy subjects. [file 1471-2164-14-265-S1.docx]

**Table S1**. Samples of myogenic cells used in the study were isolated from six healthy subjects. MB: Myoblasts; MT: Myotubes.

| Sample | Type | Subject | Tissue | Phenotype | Age | Sex | Status |
| --- | --- | --- | --- | --- | --- | --- | --- |
| 1 | MB, MT | NO40 | quadriceps | normal | 46 | M | CD56+ |
| 2 | MB, MT | NO42 | quadriceps | normal | 24 | F | CD56+ |
| 3 | MB, MT | NO44 | quadriceps | normal | 29 | M | CD56+ |
| 4 | MB, MT | NO46 | quadriceps | normal | 31 | M | CD56+ |
| 5 | MB, MT | NO47 | quadriceps | normal | 43 | M | CD56+ |
| 6 | MB, MT | NO45 | quadriceps | normal | 25 | F | CD56+ |
