# Supplementary figures and images for: Simultaneous miRNA and mRNA transcriptome profiling of human myoblasts reveals a novel set of myogenic differentiation-associated miRNAs and their target genes
Source: BMC Genomics. 2013 Apr 18;14:265. doi: 10.1186/1471-2164-14-265 (PMC3639941; doi:10.1186/1471-2164-14-265)

A

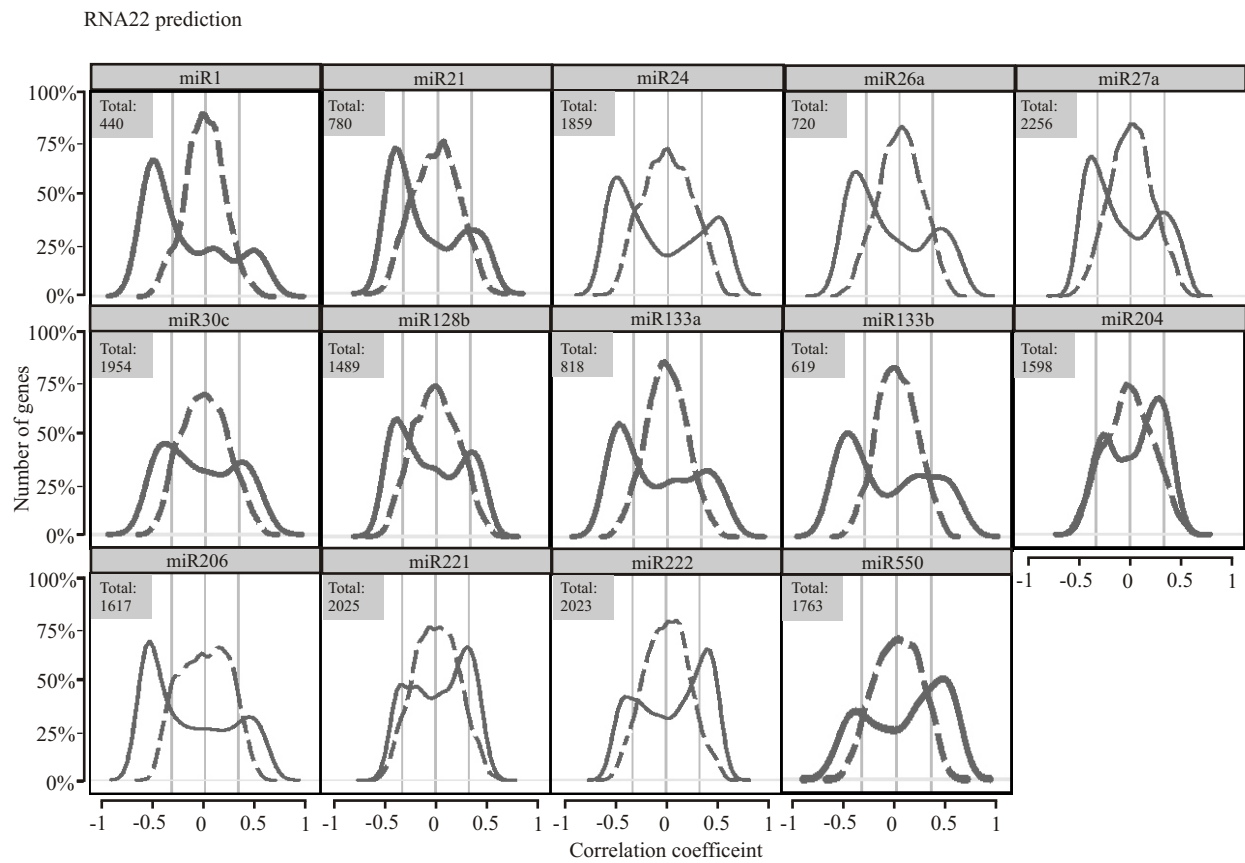

B

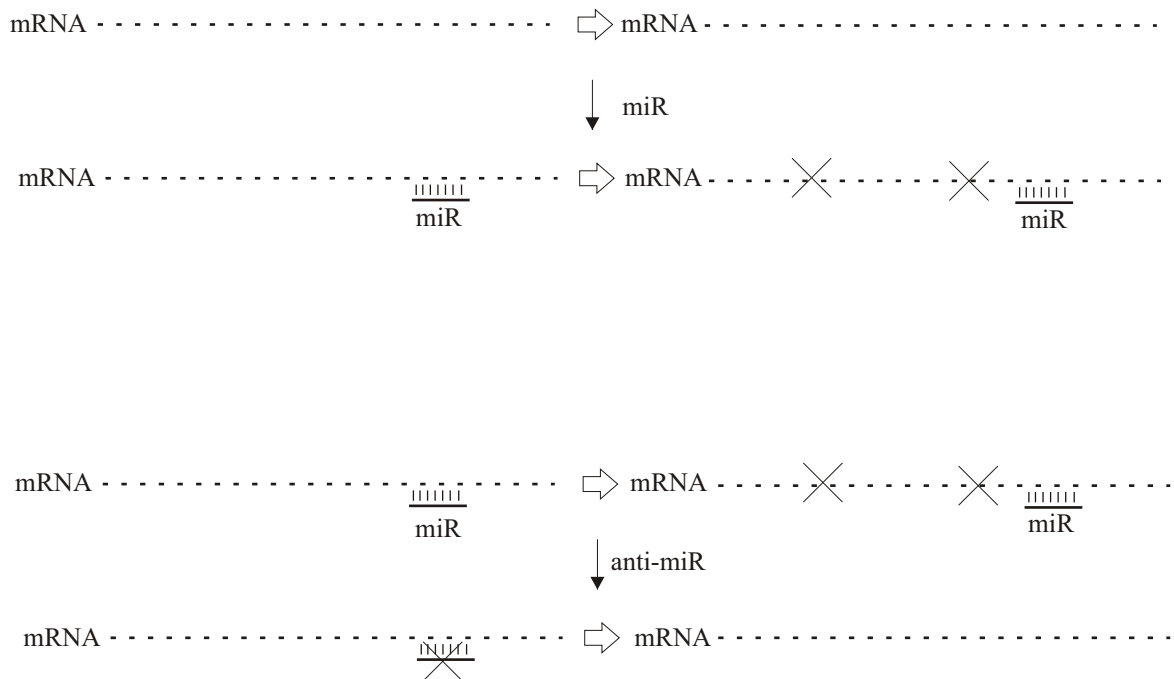

Figure S1

Supplement: Additional file 6: Figure S1 — A. Density plot of Pearson correlation coefficients between expression of miRNA and their target genes (continuous line) predicted by RNA22 algorithm and the the density plot of Pearson correlation coefficient after permutation of the list of microRNAs (dashed line). B. Schematic representation of ectopic overexpression/knockdown of miRNA in the cells that served to confirm their targets genes supported by transcriptome data. [file 1471-2164-14-265-S6.pdf]

A

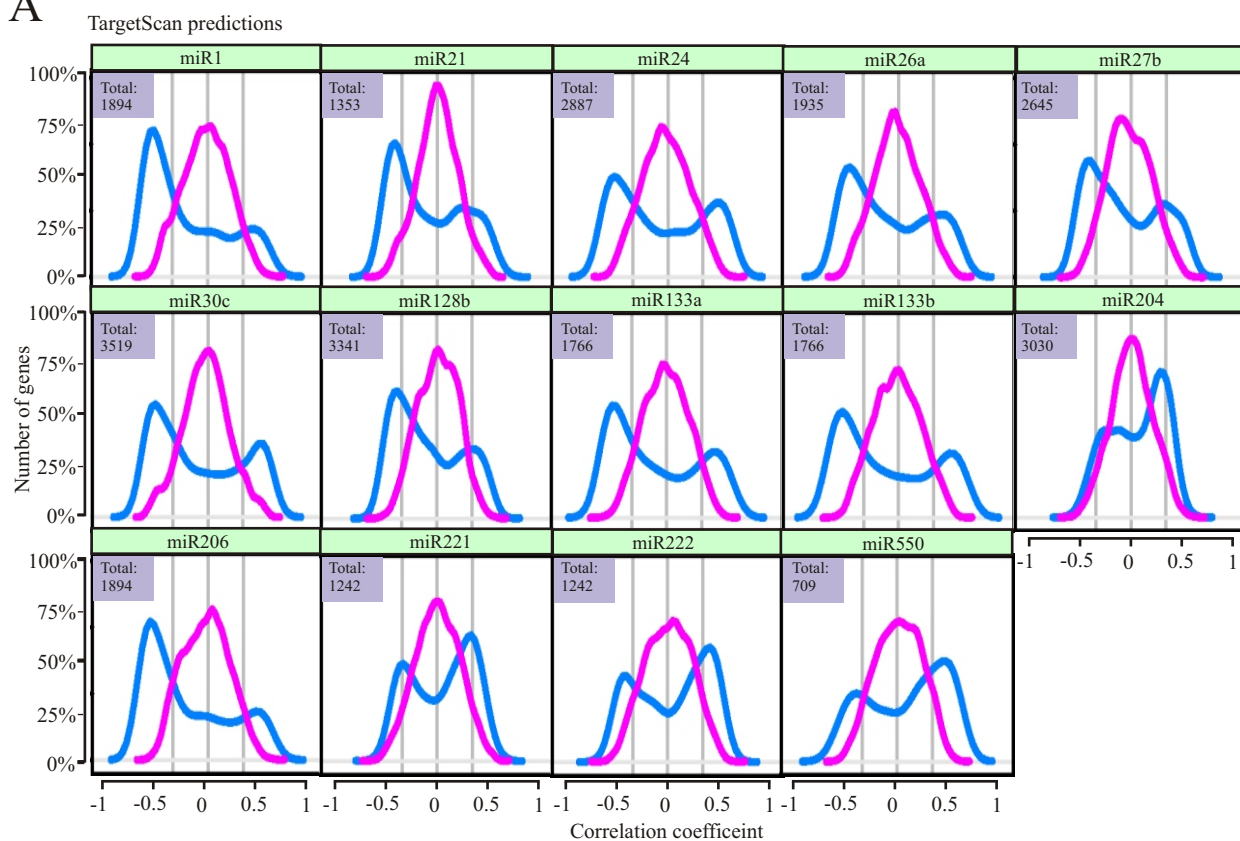

B

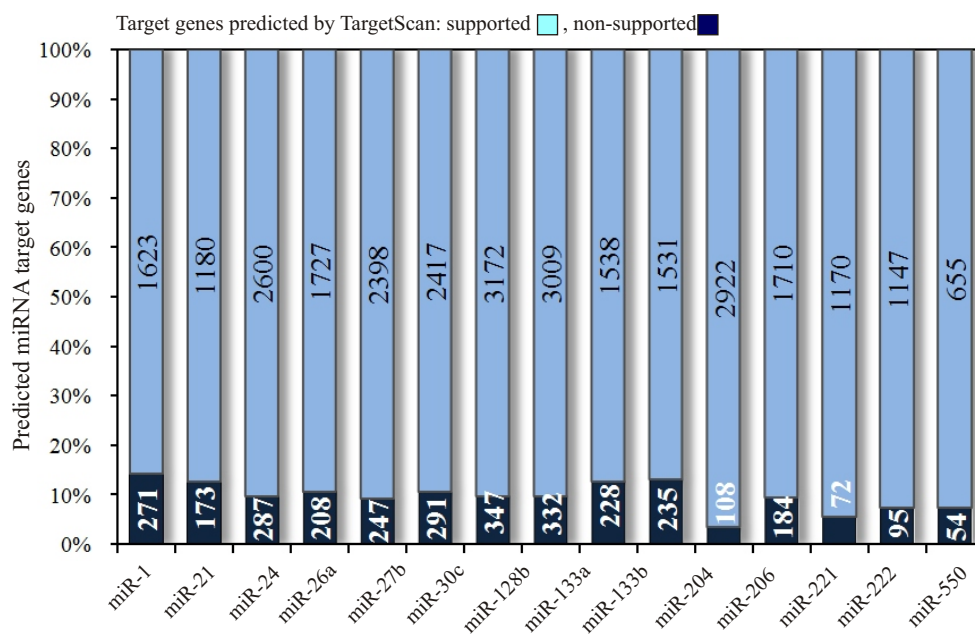

Figure S2

Supplement: Additional file 8: Figure S2A — Density plot of Pearson correlation coefficients between expression of miRNA and their target genes (blue) predicted by TargetScan algorithm and the density plot of Pearson correlation coefficient after permutation of the list of microRNAs (red). B. Diagram showing the proportion of bioinformatic predictions made by TargetScan algorithm (taken for 100%) that were supported by transcriptome profiling (black). Blue bars show unsupported predictions. [file 1471-2164-14-265-S8.pdf]
