# Supplementary material for: Simultaneous miRNA and mRNA transcriptome profiling of human myoblasts reveals a novel set of myogenic differentiation-associated miRNAs and their target genes
Source: BMC Genomics. 2013 Apr 18;14:265. doi: 10.1186/1471-2164-14-265 (PMC3639941; doi:10.1186/1471-2164-14-265)
Supplement: Additional file 9: Table S8 — Functions of miRNA target genes supported by transcriptome data. Functional classification has been performed using DAVID functional annotation tool. [file 1471-2164-14-265-S9.pdf]

miR1	Functional classes (downregulated):												
		p-value	*1,03E-17*	*1,47E-14*	*2,64E-13*	*7,26E-11*	*1,30E-09*	*1,04E-09*	*2,62E-09*	*3,36E-08*	*3,47E-08*		
Target gene name	miRNA sites	Fold change	transcription, negative reg,	cell cycle regulation	apoptosis	DNA damage response	ubiquitination/proteolysis	chromatin organization/modification	cytoskeleton organization	cell motility	protein modification	Other	Target gene description
Total: 22			*8*	*7*	*6*	*5*	*4*	*4*	*4*	*3*	*3*	*3*	
ACTR3	1	-2,04							X	X			ARP3 actin-related protein 3 homolog (yeast) (ACTR3), mRNA
ARHGEF3	1	-18,49			X								Rho guanine nucleotide exchange factor (GEF) 3 (ARHGEF3), mRNA
BAG4	1	-2,50			X								BCL2-associated athanogene 4 (BAG4), mRNA
CAND1	1	-3,36	X				X		X				cullin-associated and neddylation-dissociated 1 (CAND1), mRNA
CCND1	1	-120,05		X		X					X		cyclin D1 (CCND1), mRNA
COPA	1	-2,47										X	coatomer protein complex, subunit alpha (COPA), mRNA
DNMT1	1	-1,84	X			X		X	X		X		DNA (cytosine-5-)-methyltransferase 1 (DNMT1), mRNA
EP300	1	-2,47	X	X	X			X			X		E1A binding protein p300 (EP300), mRNA
ETS1	1	-8,26	X	X	X					X			v-ets erythroblastosis virus E26 oncogene homolog 1 (avian) (ETS1), mRNA
HLTF	1	-4,51	X				X	X					helicase-like transcription factor (HLTF), transcript variant 1, mRNA
JUB	1	-7,19		X						X			jub, ajuba homolog (Xenopus laevis) (JUB), transcript variant 1, mRNA
KCNIP3	1	-8,61	X		X								Kv channel interacting protein 3, calsenilin (KCNIP3), transcript variant 1, mRNA
NEDD9	1	-7,29		X					X				neural precursor cell expressed, developmentally down-regulated 9 (NEDD9), transcript variant 1, mRNA
NXT2	1	-5,69										X	nuclear transport factor 2-like export factor 2 (NXT2), mRNA
ORC6L	1	-11,48				X							origin recognition complex, subunit 6 like (yeast) (ORC6L), mRNA
PIK3C2A	1	-2,51										X	phosphoinositide-3-kinase, class 2, alpha polypeptide (PIK3C2A), mRNA
RAD18	1	-5,18				X	X						RAD18 homolog (S. cerevisiae) (RAD18), mRNA
RAD54B	1	-8,93		X		X							RAD54 homolog B (S. cerevisiae) (RAD54B), mRNA
RYBP	1	-5,09	X		X								RING1 and YY1 binding protein (RYBP), mRNA
SMC4	1	-25,31		X				X					structural maintenance of chromosomes 4 (SMC4), transcript variant 1, mRNA
TFE3	1	-3,35	X										transcription factor binding to IGHM enhancer 3 (TFE3), mRNA
TGFB1I1	1	-2,32					X						transforming growth factor beta 1 induced transcript 1 (TGFB1I1), transcript variant 1, mRNA

miR21			Functional classes (downregulated):											
		p-value	*1,44E-21*	*1,00E-15*	*1,97E-13*	*4,32E-11*	*1,03E-12*	*8,19E-12*	*6,47E-12*	*1,42E-12*	*2,13E-10*	*7,64E-08*		
Target gene name	miRNA sites	Fold change	transport/localization	transcription, negative reg,	reg, of kinase activity	cell cycle regulation	chromatin organization/modification	apoptosis	protein modification	NF-kB	protein complex assembly	DNA damage response	Other	Gene description
Total: 30			*12*	*8*	*7*	*6*	*6*	*6*	*5*	*5*	*5*	*4*	*4*	
ACP2	1	-2,02			X									acid phosphatase 2, lysosomal (ACP2), mRNA
AKAP7	1	-18,75	X											A kinase (PRKA) anchor protein 7 (AKAP7), transcript variant gamma, mRNA
ARHGEF3	1	-18,49						X						Rho guanine nucleotide exchange factor (GEF) 3 (ARHGEF3), mRNA
CANX	1	-4,04	X											calnexin (CANX), transcript variant 1, mRNA
CENPA	1	-190,22	X			X	X				X			centromere protein A (CENPA), transcript variant 1, mRNA
CENPH	1	-59,49				X	X				X			centromere protein H (CENPH), mRNA
CPNE3	1	-2,64	X											copine III (CPNE3), mRNA
CREBL2	1	-8,75				X								cAMP responsive element binding protein-like 2 (CREBL2), mRNA
EXT2	2	-3,51											X	exostoses (multiple) 2 (EXT2), transcript variant 1, mRNA
GADD45B	1	-10,68			X	X		X						growth arrest and DNA-damage-inducible, beta (GADD45B), mRNA
GJA1	1	-5,09	X					X	X	X	X	X		gap junction protein, alpha 1, 43kDa (connexin 43) (GJA1), mRNA
GNS	1	-2,01											X	glucosamine (N-acetyl)-6-sulfatase (Sanfilippo disease IIID) (GNS), mRNA
HP1BP3	1	-3,58					X				X			heterochromatin protein 1, binding protein 3 (HP1BP3), mRNA
MCFD2	1	-5,31	X											multiple coagulation factor deficiency 2 (MCFD2), mRNA
MXD4	2	-6,68		X										MAX dimerization protein 4 (MXD4), mRNA
NEDD4	1	-26,59	X	X					X	X		X		neural precursor cell expressed, developmentally down-regulated 4 (NEDD4), transcript variant 2, mRNA
NF2	1	-3,91		X	X				X	X				neurofibromin 2 (bilateral acoustic neuroma) (NF2), transcript variant 13, mRNA
PIK3R3	1	-2,70			X									phosphoinositide-3-kinase, regulatory subunit 3 (p55, gamma) (PIK3R3), mRNA
PLDN	1	-2,04	X											pallidin homolog (mouse) (PLDN), mRNA
PRKDC	1	-4,33		X	X		X	X				X		protein kinase, DNA-activated, catalytic polypeptide (PRKDC), mRNA
PROCR	1	-57,25											X	protein C receptor, endothelial (EPCR) (PROCR), mRNA
PSMD5	1	-2,81		X	X	X			X					proteasome (prosome, macropain) 26S subunit, non-ATPase, 5 (PSMD5), mRNA
RAB31	1	-4,72	X											RAB31, member RAS oncogene family (RAB31), mRNA
SEC22C	1	-3,73	X											SEC22 vesicle trafficking protein homolog C (S. cerevisiae) (SEC22C), transcript variant 2, mRNA
SH3RF1	2	-3,12						X		X				SH3 domain containing ring finger 1 (SH3RF1), mRNA
SLBP	1	-3,44											X	stem-loop (histone) binding protein (SLBP), mRNA
SMARCA5	1	-2,40		X			X				X			SWI/SNF related, matrix associated, actin dependent regulator of chromatin, subfamily a, member 5 (SMARC
SUZ12	1	-3,31		X			X							suppressor of zeste 12 homolog (Drosophila) (SUZ12), mRNA
SYTL4	1	-6,79	X											synaptotagmin-like 4 (granuphilin-a) (SYTL4), mRNA
THBS1	1	-73,21	X	X	X	X		X	X	X		X		thrombospondin 1 (THBS1), mRNA

miR24			Functional classes (downregulated):													
		p-value	*3,70E-23*	*4,05E-21*	*1,25E-19*	*2,04E-19*	*1,07E-18*	*2,25E-18*	*1,18E-16*	*1,15E-16*	*1,72E-13*	*3,49E-13*	*1,89E-12*	*4,54E-12*		
Target gene name	miRNA sites	Fold change	DNA damage response	transport/localization	cell cycle regulation	reg. of kinase activity	transcription, negative reg.	apoptosis	phosphorylation	NF-kB	ubiquitination/proteolysis	protein complex assembly	RNA splicing	chromatin organization/modification	Other	Gene description
Total: 72			*16*	*15*	*14*	*13*	*12*	*12*	*10*	*8*	*8*	*8*	*7*	*7*	*7*	
ADCY7	2	-5,47				X										adenylate cyclase 7 (ADCY7), mRNA
ALG2	1	-2,36		X												asparagine-linked glycosylation 2 homolog (S. cerevisiae, alpha-1,3-mannosyltransferase) (ALG2), mRNA
ANPEP	1	-168,98									X					alanyl (membrane) aminopeptidase (aminopeptidase N, aminopeptidase M, microsomal aminopeptidase, CD1
AP2B1	1	-3,31		X												adaptor-related protein complex 2, beta 1 subunit (AP2B1), transcript variant 1, mRNA
AQR	1	-2,17											X			aquarius homolog (mouse) (AQR), mRNA
ATP2B1	1	-35,02													X	ATPase, Ca++ transporting, plasma membrane 1 (ATP2B1), transcript variant 2, mRNA
AXL	1	-145,37							X							AXL receptor tyrosine kinase (AXL), transcript variant 1, mRNA
B3GNT5	1	-52,18													X	UDP-GlcNAc:betaGal beta-1,3-N-acetylglucosaminyltransferase 5 (B3GNT5), mRNA
BCL10	1	-3,26				X		X		X		X				B-cell CLL/lymphoma 10 (BCL10), mRNA
CANX	2	-4,04		X												calnexin (CANX), transcript variant 1, mRNA
CAPZA1	1	-4,63										X				capping protein (actin filament) muscle Z-line, alpha 1 (CAPZA1), mRNA
CASC3	1	-2,95		X							X		X			cancer susceptibility candidate 3 (CASC3), mRNA
CCNF	1	-10,36			X											cyclin F (CCNF), mRNA
CDC25A	1	-7,43	X		X	X			X							cell division cycle 25 homolog A (S. cerevisiae) (CDC25A), transcript variant 1, mRNA
CHIT1	1	-13,47									X					chitinase 1 (chitotriosidase) (CHIT1), mRNA
CIT	1	-14,52			X				X							citron (rho-interacting, serine/threonine kinase 21) (CIT), mRNA
CPA4	1	-9,67									X			X		carboxypeptidase A4 (CPA4), mRNA
CREBL2	2	-8,75			X											cAMP responsive element binding protein-like 2 (CREBL2), mRNA
DBF4	1	-10,20	X		X											DBF4 homolog (S. cerevisiae) (DBF4), mRNA
DBF4B	1	-4,01			X											DBF4 homolog B (S. cerevisiae) (DBF4B), transcript variant 2, mRNA
DCBLD2	1	-8,98													X	discoidin, CUB and LCCL domain containing 2 (DCBLD2), mRNA
DOCK1	2	-3,85		X				X								dedicator of cytokinesis 1 (DOCK1), mRNA
DTL	1	-36,20	X								X					denticleless homolog (Drosophila) (DTL), mRNA
EGFR	1	-7,16	X	X	X	X		X	X	X						epidermal growth factor receptor (erythroblastic leukemia viral (v-erb-b) oncogene homolog, avian) (EGFR),
EIF4H	1	-2,44													X	eukaryotic translation initiation factor 4H (EIF4H), transcript variant 1, mRNA
ENTPD2	1	-1,94													X	ectonucleoside triphosphate diphosphohydrolase 2 (ENTPD2), transcript variant 2, mRNA
FANCC	1	-4,09	X									X				Fanconi anemia, complementation group C (FANCC), mRNA
FBXL2	1	-9,22									X					F-box and leucine-rich repeat protein 2 (FBXL2), mRNA
H3F3B	1	-4,69										X		X		H3 histone, family 3B (H3.3B) (H3F3B), mRNA
HEMK1	1	-2,82	X													HemK methyltransferase family member 1 (HEMK1), mRNA
HEY1	1	-4,60					X									hairy/enhancer-of-split related with YRPW motif 1 (HEY1), transcript variant 2, mRNA
HIF1A	1	-3,61	X			X	X			X						hypoxia-inducible factor 1, alpha subunit (basic helix-loop-helix transcription factor) (HIF1A), transcript var
HPS4	2	-4,37		X												Hermansky-Pudlak syndrome 4 (HPS4), transcript variant 1, mRNA
IGFBP4	1	-10,42	X													insulin-like growth factor binding protein 4 (IGFBP4), mRNA
ILF3	1	-3,85			X		X									interleukin enhancer binding factor 3, 90kDa (ILF3), transcript variant 2, mRNA
IRAK4	1	-7,64							X							interleukin-1 receptor-associated kinase 4 (IRAK4), mRNA
KCNIP3	2	-8,61					X	X								Kv channel interacting protein 3, calsenilin (KCNIP3), transcript variant 1, mRNA
KRAS	1	-3,58	X	X		X		X		X						v-Ki-ras2 Kirsten rat sarcoma viral oncogene homolog (KRAS), transcript variant a, mRNA
MAP3K14	1	-13,92							X							mitogen-activated protein kinase kinase kinase 14 (MAP3K14), mRNA
MBD2	2	-1,74					X			X		X				methyl-CpG binding domain protein 2 (MBD2), transcript variant 1, mRNA
MECP2	1	-3,52					X									methyl CpG binding protein 2 (Rett syndrome) (MECP2), mRNA
MGAT1	1	-6,66													X	mannosyl (alpha-1,3-)-glycoprotein beta-1,2-N-acetylglucosaminyltransferase (MGAT1), mRNA
MPDU1	1	-4,86													X	mannose-P-dolichol utilization defect 1 (MPDU1), mRNA
MSH2	1	-2,22	X		X	X	X	X	X					X		mutS homolog 2, colon cancer, nonpolyposis type 1 (E. coli) (MSH2), mRNA
MXD4	2	-6,68					X									MAX dimerization protein 4 (MXD4), mRNA
NADK	1	-3,61							X							NAD kinase (NADK), mRNA
NBN	1	-4,00	X		X									X		nibrin (NBN), transcript variant 2, mRNA
NEDD9	2	-7,29			X											neural precursor cell expressed, developmentally down-regulated 9 (NEDD9), transcript variant 1, mRNA
NEK11	1	-3,51	X		X				X							NIMA (never in mitosis gene a)- related kinase 11 (NEK11), transcript variant 1, mRNA
NGFR	2	-325,61	X		X			X			X					nerve growth factor receptor (TNFR superfamily, member 16) (NGFR), mRNA
PTPLAD1	1	-7,08	X			X			X	X						protein tyrosine phosphatase-like A domain containing 1 (PTPLAD1), mRNA
RAB1B	1	-1,56		X												RAB1B, member RAS oncogene family (RAB1B), mRNA
RAB5C	1	-2,43		X												RAB5C, member RAS oncogene family (RAB5C), transcript variant 1, mRNA
RBM15B	1	-3,07		X			X						X			RNA binding motif protein 15B (RBM15B), mRNA
RFX5	1	-7,36					X									regulatory factor X, 5 (influences HLA class II expression) (RFX5), transcript variant 1, mRNA
RHOBTB3	2	-2,54		X												Rho-related BTB domain containing 3 (RHOBTB3), mRNA
SFRS7	1	-6,90											X			splicing factor, arginine/serine-rich 7, 35kDa (SFRS7), mRNA
SFRS9	1	-2,62										X	X			splicing factor, arginine/serine-rich 9 (SFRS9), mRNA
SLBP	1	-3,44											X			stem-loop (histone) binding protein (SLBP), mRNA
SMARCA5	1	-2,40					X					X		X		SWI/SNF related, matrix associated, actin dependent regulator of chromatin, subfamily a, member 5 (SMARC
SMC1A	1	-3,82	X		X								X	X		structural maintenance of chromosomes 1A (SMC1A), mRNA
SNAP23	1	-2,56		X												synaptosomal-associated protein, 23kDa (SNAP23), transcript variant 1, mRNA
SON	2	-2,12						X								SON DNA binding protein (SON), transcript variant b, mRNA
SPRY4	1	-3,32				X										sprouty homolog 4 (Drosophila) (SPRY4), mRNA
SSR1	1	-3,14		X												signal sequence receptor, alpha (translocon-associated protein alpha) (SSR1), mRNA
THY1	1	-18,39				X										Thy-1 cell surface antigen (THY1), mRNA

TLX2	1	-6,43														T-cell leukemia homeobox 2 (TLX2), mRNA
TOP2A	1	-463,68	X					X						X		topoisomerase (DNA) II alpha 170kDa (TOP2A), mRNA
TRAF7	1	-2,60				X		X		X	X					TNF receptor-associated factor 7 (TRAF7), transcript variant 1, mRNA
TWSG1	1	-3,49								X						twisted gastrulation homolog 1 (Drosophila) (TWSG1), mRNA
VAV2	1	-2,84				X		X								vav 2 oncogene (VAV2), mRNA
YWHAB	1	-1,98		X		X	X	X				X				tyrosine 3-monooxygenase/tryptophan 5-monooxygenase activation protein, beta polypeptide (YWHAB), tra

miR26a			Functional classes (downregulated):												
		p-value	*2,67E-11*	*1,39E-09*	*2,95E-08*	*3,62E-06*	*1,52E-07*	*9,67E-07*	*1,24E-06*	*2,82E-06*	*3,54E-06*	*2,60E-05*	*6,71E-05*		
Target gene name	miRNA sites	Fold change	cell cycle regulation	transport/localization	apoptosis	DNA damage response	protein modification	phosphorylation	protein complex assembly	reg, of kinase activity	M-phase	nucleotide biosynthesis	RNA splicing	Other	Gene description
Total: 20			*6*	*5*	*4*	*3*	*3*	*3*	*3*	*3*	*2*	*2*	*2*	*2*	
AP2B1	2	-3,31		X											adaptor-related protein complex 2, beta 1 subunit (AP2B1), transcript variant 1, mRNA
ARPC3	1	-3,20												X	actin related protein 2/3 complex, subunit 3, 21kDa (ARPC3), mRNA
CNP	1	-2,26										X			2',3'-cyclic nucleotide 3' phosphodiesterase (CNP), mRNA
CUL4B	1	-9,63	X			X									cullin 4B (CUL4B), mRNA
DBF4B	1	-4,01	X												DBF4 homolog B (S. cerevisiae) (DBF4B), transcript variant 2, mRNA
DHFR	1	-22,32										X			dihydrofolate reductase (DHFR), mRNA
EFCAB4B	1	-4,14		X											EF-hand calcium binding domain 4B (EFCAB4B), mRNA
FANCA	1	-16,65	X			X			X		X				Fanconi anemia, complementation group A (FANCA), transcript variant 1, mRNA
FSD1	1	-5,26	X												fibronectin type III and SPRY domain containing 1 (FSD1), mRNA
HIP1	1	-11,44		X	X				X	X					huntingtin interacting protein 1 (HIP1), mRNA
PPM1M	1	-2,66						X							protein phosphatase 1M (PP2C domain containing) (PPM1M), mRNA
PRKCD	1	-3,22					X	X		X					protein kinase C, delta (PRKCD), transcript variant 1, mRNA
QKI	1	-2,48		X			X						X		quaking homolog, KH domain RNA binding (mouse) (QKI), transcript variant 1, mRNA
RAB1B	1	-1,56		X											RAB1B, member RAS oncogene family (RAB1B), mRNA
RAD51	1	-42,86	X			X			X		X				RAD51 homolog (RecA homolog, E. coli) (S. cerevisiae) (RAD51), transcript variant 1, mRNA
RYBP	1	-5,09			X										RING1 and YY1 binding protein (RYBP), mRNA
STK39	1	-14,71						X							serine threonine kinase 39 (STE20/SPS1 homolog, yeast) (STK39), mRNA
SUMO3	1	-3,94												X	SMT3 suppressor of mif two 3 homolog 3 (S. cerevisiae) (SUMO3), mRNA
TARDBP	1	-1,59	X		X								X		TAR DNA binding protein (TARDBP), mRNA
TLR4	1	-18,67			X		X			X					toll-like receptor 4 (TLR4), mRNA

miR27a	Functional classes (downregulated):														
		p-value	*4,62E-30*	*1,85E-28*	*1,91E-26*	*2,38E-21*	*2,18E-19*	*1,53E-16*	*1,09E-17*	*1,18E-14*	*9,25E-17*	*4,23E-15*	*2,74E-11*		
Target gene name	miRNA sites	Fold change	transport/localization	apoptosis	reg, of kinase activity	protein complex assembly	cytoskeleton organization	DNA damage response	transcription, negative reg,	cell cycle regulation	cell motility	nucleotide biosynthesis	phosphorylation	Other	Gene description
Total: 89			*23*	*20*	*19*	*14*	*13*	*12*	*12*	*11*	*9*	*8*	*7*	*10*	
ACP2	1	-2,02			X										acid phosphatase 2, lysosomal (ACP2), mRNA
ADCY7	1	-5,47			X							X			adenylate cyclase 7 (ADCY7), mRNA
ADRB2	1	-36,72	X	X	X	X		X	X						adrenergic, beta-2-, receptor, surface (ADRB2), mRNA
ALG1	1	-2,63												X	asparagine-linked glycosylation 1 homolog (S. cerevisiae, beta-1,4-mannosyltransferase) (ALG1), mRNA
AMPD2	1	-2,27										X			adenosine monophosphate deaminase 2 (isoform L) (AMPD2), transcript variant 1, mRNA
AMPH	1	-5,38	X												amphiphysin (Stiff-Man syndrome with breast cancer 128kDa autoantigen) (AMPH), transcript variant 1, mR
ANXA5	1	-2,89		X	X	X									annexin A5 (ANXA5), mRNA
AQP2	1	-2,63						X							aquaporin 2 (collecting duct) (AQP2), mRNA
ARF3	1	-6,21	X												ADP-ribosylation factor 3 (ARF3), mRNA
ARFIP2	1	-2,60					X				X				ADP-ribosylation factor interacting protein 2 (arfaptin 2) (ARFIP2), mRNA
ARHGAP26	1	-3,38					X								Rho GTPase activating protein 26 (ARHGAP26), mRNA
ATP11C	1	-9,45										X			ATPase, Class VI, type 11C (ATP11C), transcript variant 2, mRNA
ATP2B1	1	-35,02										X			ATPase, Ca++ transporting, plasma membrane 1 (ATP2B1), transcript variant 2, mRNA
ATP2B4	1	-5,46										X			ATPase, Ca++ transporting, plasma membrane 4 (ATP2B4), transcript variant 1, mRNA
ATP8A2	1	-5,90										X			ATPase, aminophospholipid transporter-like, Class I, type 8A, member 2 (ATP8A2), mRNA
BAG5	1	-1,99		X											BCL2-associated athanogene 5 (BAG5), transcript variant 1, mRNA
BUB1B	1	-493,74		X			X	X	X	X					BUB1 budding uninhibited by benzimidazoles 1 homolog beta (yeast) (BUB1B), mRNA
CALR	1	-4,96	X	X		X	X		X	X					calreticulin (CALR), mRNA
CAPZA1	1	-4,63				X	X				X				capping protein (actin filament) muscle Z-line, alpha 1 (CAPZA1), mRNA
CASC3	1	-2,95	X												cancer susceptibility candidate 3 (CASC3), mRNA
CDC6	1	-9,90			X			X	X	X					cell division cycle 6 homolog (S. cerevisiae) (CDC6), mRNA
CKS1B	1	-24,96			X					X					CDC28 protein kinase regulatory subunit 1B (CKS1B), mRNA
CLOCK	1	-1,68						X	X						clock homolog (mouse) (CLOCK), mRNA
CSE1L	1	-2,58	X	X		X									CSE1 chromosome segregation 1-like (yeast) (CSE1L), mRNA
CTNNB1	1	-8,78	X	X	X	X	X		X	X					catenin (cadherin-associated protein), beta 1, 88kDa (CTNNB1), mRNA
EPHA2	1	-8,22											X		EPH receptor A2 (EPHA2), mRNA
ETS1	2	-8,26		X					X	X	X				v-ets erythroblastosis virus E26 oncogene homolog 1 (avian) (ETS1), mRNA
FANCC	1	-4,09				X		X							Fanconi anemia, complementation group C (FANCC), mRNA
FZD1	1	-79,02							X						frizzled homolog 1 (Drosophila) (FZD1), mRNA
GCA	1	-7,82	X												grancalcin, EF-hand calcium binding protein (GCA), mRNA
GM2A	1	-4,39			X										GM2 ganglioside activator (GM2A), mRNA
GNS	1	-2,01												X	glucosamine (N-acetyl)-6-sulfatase (Sanfilippo disease IIID) (GNS), mRNA
H2AFZ	1	-4,61				X									H2A histone family, member Z (H2AFZ), mRNA
HP1BP3	1	-3,58				X									heterochromatin protein 1, binding protein 3 (HP1BP3), mRNA
HSP90AA1	1	-2,29	X		X	X									heat shock protein 90kDa alpha (cytosolic), class A member 1 (HSP90AA1), transcript variant 2, mRNA
HSPD1	1	-2,36		X	X	X		X							heat shock 60kDa protein 1 (chaperonin) (HSPD1), nuclear gene encoding mitochondrial protein, transcript v
IGF2BP3	1	-3,22							X						insulin-like growth factor 2 mRNA binding protein 3 (IGF2BP3), mRNA
IGFBP4	1	-10,42						X							insulin-like growth factor binding protein 4 (IGFBP4), mRNA
ING5	1	-2,15						X							inhibitor of growth family, member 5 (ING5), mRNA
KRAS	1	-3,58	X	X	X		X	X							v-Ki-ras2 Kirsten rat sarcoma viral oncogene homolog (KRAS), transcript variant a, mRNA
LASP1	1	-13,02					X								LIM and SH3 protein 1 (LASP1), mRNA
LY86	1	-2,00		X											lymphocyte antigen 86 (LY86), mRNA
MAN2A1	1	-40,58												X	mannosidase, alpha, class 2A, member 1 (MAN2A1), mRNA
MAP3K14	1	-13,92											X		mitogen-activated protein kinase kinase kinase 14 (MAP3K14), mRNA
MET	1	-3,40			X						X		X		met proto-oncogene (hepatocyte growth factor receptor) (MET), mRNA
MYD88	1	-7,46		X	X										myeloid differentiation primary response gene (88) (MYD88), mRNA
MYH10	1	-2,95	X				X			X	X				myosin, heavy chain 10, non-muscle (MYH10), mRNA
NADK	1	-3,61										X	X		NAD kinase (NADK), mRNA
NGFR	1	-325,61		X			X	X		X	X				nerve growth factor receptor (TNFR superfamily, member 16) (NGFR), mRNA
NOLC1	1	-1,92								X					nucleolar and coiled-body phosphoprotein 1 (NOLC1), mRNA
NUDT21	2	-4,18												X	nudix (nucleoside diphosphate linked moiety X)-type motif 21 (NUDT21), mRNA
NXT2	1	-5,69	X												nuclear transport factor 2-like export factor 2 (NXT2), mRNA
OSBPL5	1	-6,32	X												oxysterol binding protein-like 5 (OSBPL5), transcript variant 1, mRNA
PIK3R3	1	-2,70			X										phosphoinositide-3-kinase, regulatory subunit 3 (p55, gamma) (PIK3R3), mRNA
PLDN	1	-2,04	X												pallidin homolog (mouse) (PLDN), mRNA
PMM2	1	-2,30												X	phosphomannomutase 2 (PMM2), mRNA
POLR2L	1	-2,77				X									polymerase (RNA) II (DNA directed) polypeptide L, 7.6kDa (POLR2L), mRNA
POM121	1	-3,83	X												POM121 membrane glycoprotein (rat) (POM121), mRNA
PPAP2B	1	-15,34									X				phosphatidic acid phosphatase type 2B (PPAP2B), transcript variant 1, mRNA
PRPF38A	1	-5,34												X	PRP38 pre-mRNA processing factor 38 (yeast) domain containing A (PRPF38A), transcript variant 1, mRNA
PTPN9	1	-6,10											X		protein tyrosine phosphatase, non-receptor type 9 (PTPN9), mRNA
PUS7L	1	-1,82												X	pseudouridylate synthase 7 homolog (S. cerevisiae)-like (PUS7L), mRNA
RAB8A	1	-3,01	X												RAB8A, member RAS oncogene family (RAB8A), mRNA
RBM15B	3	-3,07	X						X						RNA binding motif protein 15B (RBM15B), mRNA
ROBO4	1	-37,10												X	roundabout homolog 4, magic roundabout (Drosophila) (ROBO4), mRNA
RYBP	2	-5,09		X					X						RING1 and YY1 binding protein (RYBP), mRNA
SDC1	1	-10,82			X										syndecan 1 (SDC1), transcript variant 1, mRNA
SEC22A	2	-1,77	X												SEC22 vesicle trafficking protein homolog A (S. cerevisiae) (SEC22A), mRNA
SH3GL1	1	-11,32	X												SH3-domain GRB2-like 1 (SH3GL1), mRNA

SIM2	1	-2,55							X						single-minded homolog 2 (Drosophila) (SIM2), transcript variant SIM2, mRNA
SLC1A3	1	-39,30	X					X				X			solute carrier family 1 (glial high affinity glutamate transporter), member 3 (SLC1A3), mRNA
SLC22A4	1	-9,49												X	solute carrier family 22 (organic cation transporter), member 4 (SLC22A4), mRNA
SLC23A2	1	-2,33	X												solute carrier family 23 (nucleobase transporters), member 2 (SLC23A2), transcript variant 2, mRNA
STK35	1	-1,61											X		serine/threonine kinase 35 (STK35), mRNA
STXBP4	1	-7,21	X		X										syntaxin binding protein 4 (STXBP4), mRNA
TACC1	1	-6,34								X					transforming, acidic coiled-coil containing protein 1 (TACC1), mRNA
THY1	1	-18,39			X		X								Thy-1 cell surface antigen (THY1), mRNA
TLX2	1	-6,43					X								T-cell leukemia homeobox 2 (TLX2), mRNA
TMEM102	1	-2,50		X	X										transmembrane protein 102 (TMEM102), mRNA
TNFSF12	1	-5,47		X							X				tumor necrosis factor (ligand) superfamily, member 12 (TNFSF12), mRNA
TPX2	1	-113,94								X					TPX2, microtubule-associated, homolog (Xenopus laevis) (TPX2), mRNA
TRAF7	1	-2,60		X	X										TNF receptor-associated factor 7 (TRAF7), transcript variant 1, mRNA
TRIM4	1	-5,16				X									tripartite motif-containing 4 (TRIM4), transcript variant alpha, mRNA
TRIO	1	-1,98		X									X		triple functional domain (PTPRF interacting) (TRIO), mRNA
TTLL3	1	-2,57				X	X								tubulin tyrosine ligase-like family, member 3 (TTLL3), transcript variant 2, mRNA
UBE2L6	1	-4,27												X	ubiquitin-conjugating enzyme E2L 6 (UBE2L6), transcript variant 2, mRNA
VCL	1	-2,85									X				vinculin (VCL), transcript variant 1, mRNA
YWHAZ	1	-4,72	X	X											tyrosine 3-monooxygenase/tryptophan 5-monooxygenase activation protein, zeta polypeptide (YWHAZ), tran
ZNF443	1	-2,16		X											zinc finger protein 443 (ZNF443), mRNA

miR30c		Functional classes (downregulated):												
		p-value	*3,75E-21*	*1,02E-17*	*5,17E-14*	*4,18E-14*	*1,05E-15*	*6,83E-15*	*9,06E-12*	*2,56E-12*	*6,03E-10*	*5,06E-10*		
Target gene name	miRNA sites	Fold change	phosphorylation	reg. of kinase activity	cell cycle regulation	transport/localization	cytoskeleton organization	ubiquitination/proteolysis	DNA damage response	apoptosis	cell motility	nucleotide biosynthesis	Other	Gene description
Total: 66			*13*	*12*	*10*	*10*	*10*	*9*	*8*	*8*	*5*	*5*	*9*	
ANKRD27	1	-2,21				X								ankyrin repeat domain 27 (VPS9 domain) (ANKRD27), mRNA
ARFIP2	1	-2,60					X				X			ADP-ribosylation factor interacting protein 2 (arfaptin 2) (ARFIP2), mRNA
ARL3	1	-5,12			X									ADP-ribosylation factor-like 3 (ARL3), mRNA
ATP2B1	1	-35,02										X		ATPase, Ca++ transporting, plasma membrane 1 (ATP2B1), transcript variant 2, mRNA
ATP9B	1	-3,84										X		ATPase, Class II, type 9B (ATP9B), mRNA
B3GNT5	1	-52,18											X	UDP-GlcNAc:betaGal beta-1,3-N-acetylglucosaminyltransferase 5 (B3GNT5), mRNA
C1RL	1	-4,54						X						complement component 1, r subcomponent-like (C1RL), mRNA
C5	1	-7,83	X	X				X		X				complement component 5 (C5), mRNA
CALM3	1	-4,02		X	X									calmodulin 3 (phosphorylase kinase, delta) (CALM3), mRNA
CAV2	1	-5,42	X	X	X	X	X				X			caveolin 2 (CAV2), transcript variant 1, mRNA
CDCA5	1	-75,45			X	X								cell division cycle associated 5 (CDCA5), mRNA
CNP	1	-2,26					X					X		2',3'-cyclic nucleotide 3' phosphodiesterase (CNP), mRNA
CNTN2	1	-5,30									X			contactin 2 (axonal) (CNTN2), mRNA
COG8	1	-2,30				X								component of oligomeric golgi complex 8 (COG8), mRNA
CORO1C	1	-12,43				X								coronin, actin binding protein, 1C (CORO1C), mRNA
CTSC	1	-9,37		X				X						cathepsin C (CTSC), transcript variant 1, mRNA
DCTD	1	-2,23										X		dCMP deaminase (DCTD), transcript variant 1, mRNA
DDIT4	2	-11,46								X				DNA-damage-inducible transcript 4 (DDIT4), mRNA
DOCK7	1	-2,73		X			X							dedicator of cytokinesis 7 (DOCK7), mRNA
DTL	1	-36,20						X	X					denticleless homolog (Drosophila) (DTL), mRNA
EIF4H	1	-2,44											X	eukaryotic translation initiation factor 4H (EIF4H), transcript variant 1, mRNA
FBXO5	1	-20,02		X	X		X	X						F-box protein 5 (FBXO5), mRNA
FMOD	1	-5,76											X	fibromodulin (FMOD), mRNA
GADD45B	1	-10,68	X	X	X					X				growth arrest and DNA-damage-inducible, beta (GADD45B), mRNA
GAS2L1	1	-5,69			X									growth arrest-specific 2 like 1 (GAS2L1), transcript variant 2, mRNA
GAS8	1	-3,15									X			growth arrest-specific 8 (GAS8), mRNA
GCA	1	-7,82				X								grancalcin, EF-hand calcium binding protein (GCA), mRNA
GGCX	1	-15,63											X	gamma-glutamyl carboxylase (GGCX), mRNA
GNA12	1	-1,87											X	guanine nucleotide binding protein (G protein) alpha 12 (GNA12), mRNA
GNAI2	1	-8,61	X	X										guanine nucleotide binding protein (G protein), alpha inhibiting activity polypeptide 2 (GNAI2), mRNA
GOSR1	1	-2,86				X								golgi SNAP receptor complex member 1 (GOSR1), transcript variant 1, mRNA
HEMK1	2	-2,82							X					HemK methyltransferase family member 1 (HEMK1), mRNA
HPS4	1	-4,37				X								Hermansky-Pudlak syndrome 4 (HPS4), transcript variant 1, mRNA
LASP1	1	-13,02					X							LIM and SH3 protein 1 (LASP1), mRNA
LRPAP1	1	-2,94				X								low density lipoprotein receptor-related protein associated protein 1 (LRPAP1), mRNA
LTBR	1	-3,18								X				lymphotoxin beta receptor (TNFR superfamily, member 3) (LTBR), mRNA
MDFIC	1	-3,57	X	X					X					MyoD family inhibitor domain containing (MDFIC), mRNA
MICB	2	-5,04							X	X				MHC class I polypeptide-related sequence B (MICB), mRNA
MX1	1	-71,01								X				myxovirus (influenza virus) resistance 1, interferon-inducible protein p78 (mouse) (MX1), mRNA
MXD4	1	-6,68												MAX dimerization protein 4 (MXD4), mRNA
NEDD9	1	-7,29			X		X							neural precursor cell expressed, developmentally down-regulated 9 (NEDD9), transcript variant 1, mRNA
NUDT21	1	-4,18											X	nudix (nucleoside diphosphate linked moiety X)-type motif 21 (NUDT21), mRNA
PAN3	1	-3,00	X					X						PAN3 polyA specific ribonuclease subunit homolog (S. cerevisiae) (PAN3), mRNA
PIK3R3	2	-2,70		X										phosphoinositide-3-kinase, regulatory subunit 3 (p55, gamma) (PIK3R3), mRNA
PLS3	1	-5,94					X							plastin 3 (T isoform) (PLS3), mRNA
PNRC2	1	-2,72						X						proline-rich nuclear receptor coactivator 2 (PNRC2), mRNA
PPIA	1	-5,63							X					peptidylprolyl isomerase A (cyclophilin A) (PPIA), mRNA
PPM1F	1	-5,82	X							X				protein phosphatase 1F (PP2C domain containing) (PPM1F), mRNA
PPM1M	1	-2,66	X											protein phosphatase 1M (PP2C domain containing) (PPM1M), mRNA
PRKAA1	1	-1,93	X	X			X							protein kinase, AMP-activated, alpha 1 catalytic subunit (PRKAA1), transcript variant 2, mRNA
PRPF4B	1	-2,12	X											PRP4 pre-mRNA processing factor 4 homolog B (yeast) (PRPF4B), mRNA
PTTG1IP	1	-28,68				X								pituitary tumor-transforming 1 interacting protein (PTTG1IP), mRNA
RAD18	1	-5,18						X	X					RAD18 homolog (S. cerevisiae) (RAD18), mRNA
ROBO4	1	-37,10											X	roundabout homolog 4, magic roundabout (Drosophila) (ROBO4), mRNA
SDC1	1	-10,82		X										syndecan 1 (SDC1), transcript variant 1, mRNA
SENP1	1	-6,09						X						SUMO1/sentrin specific peptidase 1 (SENP1), mRNA
SNAI2	1	-63,66							X					snail homolog 2 (Drosophila) (SNAI2), mRNA
SS18	1	-6,34					X							synovial sarcoma translocation, chromosome 18 (SS18), transcript variant 1, mRNA
SSH3	1	-3,46	X											slingshot homolog 3 (Drosophila) (SSH3), mRNA
STK35	1	-1,61	X											serine/threonine kinase 35 (STK35), mRNA
TRIM4	1	-5,16											X	tripartite motif-containing 4 (TRIM4), transcript variant alpha, mRNA
TRRAP	1	-2,11			X				X					transformation/transcription domain-associated protein (TRRAP), mRNA
TWIST1	1	-43,09								X	X			twist homolog 1 (acrocephalosyndactyly 3; Saethre-Chotzen syndrome) (Drosophila) (TWIST1), mRNA
TWSG1	2	-3,49											X	twisted gastrulation homolog 1 (Drosophila) (TWSG1), mRNA
UHMK1	1	-5,11	X		X									U2AF homology motif (UHM) kinase 1 (UHMK1), mRNA
UMPS	1	-11,38										X		uridine monophosphate synthetase (orotate phosphoribosyl transferase and orotidine-5'-decarboxylase) (UMPS

**miR128b**	Functional classes (downregulated):														
		p-value	*3,30E-17*	*2,76E-15*	*1,18E-13*	*7,47E-11*	*3,47E-09*	*1,46E-08*	*1,42E-08*	*1,49E-08*	*1,75E-07*	*4,21E-07*	*7,30E-07*		
Target gene name	miRNA sites	Fold change	DNA damage response	transport/localization	cytoskeleton organization	cell cycle regulation	ubiquitination/proteolysis	apoptosis	cell motility	protein modification	phosphorylation	transcription, negative reg,	reg, of kinase activity	Other	Gene description
Total: 37			*11*	*10*	*8*	*7*	*5*	*5*	*4*	*4*	*4*	*4*	*4*	*2*	
AQP2	1	-2,63	X												aquaporin 2 (collecting duct) (AQP2), mRNA
ARFIP2	1	-2,60			X				X						ADP-ribosylation factor interacting protein 2 (arfaptin 2) (ARFIP2), mRNA
ARHGAP26	1	-3,38			X										Rho GTPase activating protein 26 (ARHGAP26), mRNA
ARPC4	1	-1,97			X										actin related protein 2/3 complex, subunit 4, 20kDa (ARPC4), transcript variant 1, mRNA
AXL	1	-145,37									X				AXL receptor tyrosine kinase (AXL), transcript variant 1, mRNA
BUB1B	1	-493,74	X		X	X	X	X		X		X			BUB1 budding uninhibited by benzimidazoles 1 homolog beta (yeast) (BUB1B), mRNA
CAV1	1	-29,48	X	X	X					X		X	X		caveolin 1, caveolae protein, 22kDa (CAV1), mRNA
CDCA5	1	-75,45		X		X									cell division cycle associated 5 (CDCA5), mRNA
CEP76	1	-2,67			X	X									centrosomal protein 76kDa (CEP76), mRNA
CLOCK	1	-1,68	X									X			clock homolog (mouse) (CLOCK), mRNA
DTYMK	1	-21,07				X									deoxythymidylate kinase (thymidylate kinase) (DTYMK), mRNA
EHD1	1	-5,43		X											EH-domain containing 1 (EHD1), mRNA
ELMOD1	1	-17,05		X											ELMO/CED-12 domain containing 1 (ELMOD1), mRNA
EML4	1	-8,63			X	X									echinoderm microtubule associated protein like 4 (EML4), mRNA
EPHA2	1	-8,22									X				EPH receptor A2 (EPHA2), mRNA
GCA	1	-7,82		X											grancalcin, EF-hand calcium binding protein (GCA), mRNA
IGFBP4	1	-10,42	X												insulin-like growth factor binding protein 4 (IGFBP4), mRNA
ING5	2	-2,15	X												inhibitor of growth family, member 5 (ING5), mRNA
NAGPA	1	-3,15		X											N-acetylglucosamine-1-phosphodiester alpha-N-acetylglucosaminidase (NAGPA), mRNA
NCAPD2	1	-24,05				X									non-SMC condensin I complex, subunit D2 (NCAPD2), mRNA
NGFR	1	-325,61	X		X	X	X	X	X						nerve growth factor receptor (TNFR superfamily, member 16) (NGFR), mRNA
PARP3	1	-2,93	X												poly (ADP-ribose) polymerase family, member 3 (PARP3), transcript variant 3, mRNA
PROCR	1	-57,25												X	protein C receptor, endothelial (EPCR) (PROCR), mRNA
PTPN9	1	-6,10									X				protein tyrosine phosphatase, non-receptor type 9 (PTPN9), mRNA
PXDN	1	-9,44	X												peroxidasin homolog (Drosophila) (PXDN), mRNA
QKI	1	-2,48		X						X					quaking homolog, KH domain RNA binding (mouse) (QKI), transcript variant 1, mRNA
QTRTD1	1	-3,54												X	queuine tRNA-ribosyltransferase domain containing 1 (QTRTD1), mRNA
RBM15B	2	-3,07		X								X			RNA binding motif protein 15B (RBM15B), mRNA
RFC2	1	-2,07	X												replication factor C (activator 1) 2, 40kDa (RFC2), transcript variant 1, mRNA
SLC1A3	1	-39,30	X	X											solute carrier family 1 (glial high affinity glutamate transporter), member 3 (SLC1A3), mRNA
STK35	1	-1,61									X				serine/threonine kinase 35 (STK35), mRNA
TRAF7	2	-2,60					X	X					X		TNF receptor-associated factor 7 (TRAF7), transcript variant 1, mRNA
USP33	1	-3,27					X								ubiquitin specific peptidase 33 (USP33), transcript variant 1, mRNA
USP4	1	-1,66					X								ubiquitin specific peptidase 4 (proto-oncogene) (USP4), transcript variant 1, mRNA
VAV2	2	-2,84						X	X				X		vav 2 oncogene (VAV2), mRNA
VEGFC	1	-3,73							X	X			X		vascular endothelial growth factor C (VEGFC), mRNA
YWHAZ	1	-4,72		X				X							tyrosine 3-monooxygenase/tryptophan 5-monooxygenase activation protein, zeta polypeptide (YWHAZ), tran

**miR133a**	Functional classes (downregulated):											
		p-value	*3,60E-16*	*5,30E-06*	*1,42E-06*	*8,06E-07*	*1,39E-06*	*3,86E-04*	*1,96E-04*	*2,22E-04*		
Target gene name	miRNA sites	Fold change	transport/localization	DNA damage response	phosphorylation	RNA splicing	ubiquitination/proteolysis	cell cycle regulation	transcription, negative reg,	apoptosis	Other	Gene description
Total: 19			*9*	*3*	*3*	*3*	*3*	*2*	*2*	*2*	*2*	
BTBD12	1	-4,11		X			X					BTB (POZ) domain containing 12 (BTBD12), mRNA
CASC3	1	-2,95	X			X	X					cancer susceptibility candidate 3 (CASC3), mRNA
CLTA	1	-4,20	X									clathrin, light chain (Lca) (CLTA), transcript variant 2, mRNA
EPHA2	1	-8,22			X							EPH receptor A2 (EPHA2), mRNA
HIP1	1	-11,44	X							X		huntingtin interacting protein 1 (HIP1), mRNA
LASP1	1	-13,02									X	LIM and SH3 protein 1 (LASP1), mRNA
LIN7C	1	-2,41	X									lin-7 homolog C (C. elegans) (LIN7C), mRNA
MDC1	1	-3,79		X				X				mediator of DNA damage checkpoint 1 (MDC1), mRNA
PRPF38A	2	-5,34				X						PRP38 pre-mRNA processing factor 38 (yeast) domain containing A (PRPF38A), transcript variant 1, mRNA
PTPN18	2	-3,43			X							protein tyrosine phosphatase, non-receptor type 18 (brain-derived) (PTPN18), mRNA
RBM15B	1	-3,07	X			X			X			RNA binding motif protein 15B (RBM15B), mRNA
RPA1	1	-5,27		X			X	X				replication protein A1, 70kDa (RPA1), mRNA
SCLT1	1	-1,85	X									sodium channel and clathrin linker 1 (SCLT1), mRNA
SNX6	1	-3,25	X						X			sorting nexin 6 (SNX6), transcript variant 1, mRNA
SON	1	-2,12								X		SON DNA binding protein (SON), transcript variant b, mRNA
SSR2	1	-5,81	X									signal sequence receptor, beta (translocon-associated protein beta) (SSR2), mRNA
SYNPO	1	-28,34									X	synaptopodin (SYNPO), mRNA
SYTL3	1	-6,86	X									synaptotagmin-like 3 (SYTL3), mRNA
VRK3	1	-2,21			X							vaccinia related kinase 3 (VRK3), transcript variant 1, mRNA

miR133b	Functional classes (downregulated):										
		p-value	*9,50E-12*	*3,28E-06*	*2,30E-06*	*1,02E-06*	*7,23E-05*	*4,96E-05*	*7,14E-05*		
Target gene name	miRNA sites	Fold change	transport/localization	cell cycle regulation	DNA damage response	cytoskeleton organization	phosphorylation	RNA splicing	ubiquitination/proteolysis	Other	Gene description
Total: 16			*6*	*3*	*3*	*3*	*2*	*2*	*2*	*2*	
BTBD12	1	-4,11			X				X		BTB (POZ) domain containing 12 (BTBD12), mRNA
CLTA	1	-4,20	X								clathrin, light chain (Lca) (CLTA), transcript variant 2, mRNA
EPHA2	1	-8,22					X				EPH receptor A2 (EPHA2), mRNA
LASP1	1	-13,02				X					LIM and SH3 protein 1 (LASP1), mRNA
MDC1	1	-3,79		X	X						mediator of DNA damage checkpoint 1 (MDC1), mRNA
MTPN	1	-3,28								X	myotrophin (MTPN), mRNA
NEDD9	1	-7,29		X		X					neural precursor cell expressed, developmentally down-regulated 9 (NEDD9), transcript variant 1, mRNA
PRPF38A	1	-5,34						X			PRP38 pre-mRNA processing factor 38 (yeast) domain containing A (PRPF38A), transcript variant 1, mRNA
RBM15B	1	-3,07	X					X			RNA binding motif protein 15B (RBM15B), mRNA
RPA1	1	-5,27		X	X				X		replication protein A1, 70kDa (RPA1), mRNA
SNX6	1	-3,25	X								sorting nexin 6 (SNX6), transcript variant 1, mRNA
SON	1	-2,12								X	SON DNA binding protein (SON), transcript variant b, mRNA
SSR2	1	-5,81	X								signal sequence receptor, beta (translocon-associated protein beta) (SSR2), mRNA
SYTL3	1	-6,86	X								synaptotagmin-like 3 (SYTL3), mRNA
TLN2	1	-2,95	X			X					talin 2 (TLN2), mRNA
VRK3	1	-2,21					X				vaccinia related kinase 3 (VRK3), transcript variant 1, mRNA

**miR204**	Functional classes (upregulated):											
		p-value	*1,62E-06*	*1,24E-06*	*4,59E-06*	*2,00E-05*	*5,01E-05*	*3,52E-05*	*7,68E-06*		
Target gene name	miRNA sites	Fold change	transcription regulation	transcription, pos, reg	ubiquitination/proteolysis	cellular respiration	transport/localization	apoptosis	cytoskeleton organization	Other	Gene description
Total: 14			*5*	*4*	*4*	*3*	*3*	*3*	*3*	*0*	
ABCD1	1	4,31					X				ATP-binding cassette, sub-family D (ALD), member 1 (ABCD1), mRNA
CIC	1	2,62	X								capicua homolog (Drosophila) (CIC), mRNA
CP110	1	3,26							X		CP110 protein (CP110), mRNA
ERGIC1	1	5,71					X				endoplasmic reticulum-golgi intermediate compartment (ERGIC) 1 (ERGIC1), transcript variant 1, mRNA
GPD1L	1	8,59				X					glycerol-3-phosphate dehydrogenase 1-like (GPD1L), mRNA
IGFBP5	1	6476,31			X						insulin-like growth factor binding protein 5 (IGFBP5), mRNA
PPARGC1A	1	95,52	X	X	X	X			X		peroxisome proliferator-activated receptor gamma, coactivator 1 alpha (PPARGC1A), mRNA
RB1CC1	1	3,37	X	X	X			X			RB1-inducible coiled-coil 1 (RB1CC1), mRNA
RRM2B	1	8,04				X		X			ribonucleotide reductase M2 B (TP53 inducible) (RRM2B), mRNA
STAT5B	2	10,87	X	X				X			signal transducer and activator of transcription 5B (STAT5B), mRNA
STX1A	1	4,00					X				syntaxin 1A (brain) (STX1A), mRNA
TEF	1	3,13	X	X							thyrotrophic embryonic factor (TEF), mRNA
TTL	1	18,69							X		tubulin tyrosine ligase (TTL), mRNA
USP30	1	3,15			X						ubiquitin specific peptidase 30 (USP30), mRNA

miR206	Functional classes (downregulated):															
		p-value	*2,48E-30*	*2,74E-28*	*1,37E-25*	*2,67E-23*	*1,32E-20*	*1,05E-18*	*4,16E-18*	*3,86E-20*	*2,92E-17*	*4,62E-18*	*1,24E-15*	*7,43E-16*		
Target gene name	miRNA sites	Fold change	transcription, negative reg,	apoptosis	DNA damage response	cell cycle regulation	transport/localization	reg, of kinase activity	cytoskeleton organization	nucleotide biosynthesis	phosphorylation	cell motility	ubiquitination/proteolysis	chromatin organization/modification	Other	Gene description
Total: 90			*19*	*18*	*17*	*16*	*14*	*12*	*11*	*10*	*10*	*9*	*9*	*9*	*9*	
ACTL6A	1	-4,17												X		actin-like 6A (ACTL6A), transcript variant 3, mRNA
ACTR3	1	-2,04						X	X			X				ARP3 actin-related protein 3 homolog (yeast) (ACTR3), mRNA
AK5	1	-14,69								X						adenylate kinase 5 (AK5), transcript variant 1, mRNA
AP1B1	1	-1,81					X									adaptor-related protein complex 1, beta 1 subunit (AP1B1), transcript variant 1, mRNA
API5	1	-3,47		X												apoptosis inhibitor 5 (API5), mRNA
ARHGAP26	1	-3,38							X							Rho GTPase activating protein 26 (ARHGAP26), mRNA
ARHGEF3	1	-18,49		X												Rho guanine nucleotide exchange factor (GEF) 3 (ARHGEF3), mRNA
ATP2B1	1	-35,02								X						ATPase, Ca++ transporting, plasma membrane 1 (ATP2B1), transcript variant 2, mRNA
ATP9B	1	-3,84								X						ATPase, Class II, type 9B (ATP9B), mRNA
BAG4	1	-2,50		X												BCL2-associated athanogene 4 (BAG4), mRNA
BCL2L11	1	-3,80		X												BCL2-like 11 (apoptosis facilitator) (BCL2L11), transcript variant 1, mRNA
CAND1	2	-3,36	X						X				X			cullin-associated and neddylation-dissociated 1 (CAND1), mRNA
CCND1	1	-120,05			X	X		X			X					cyclin D1 (CCND1), mRNA
CD302	1	-4,09					X									CD302 molecule (CD302), mRNA
CECR1	1	-2,00								X						cat eye syndrome chromosome region, candidate 1 (CECR1), transcript variant 1, mRNA
CLP1	1	-2,26	X													CLP1, cleavage and polyadenylation factor I subunit, homolog (S. cerevisiae) (CLP1), mRNA
COL3A1	1	-7,10			X			X								collagen, type III, alpha 1 (Ehlers-Danlos syndrome type IV, autosomal dominant) (COL3A1), mRNA
COPA	1	-2,47					X									coatomer protein complex, subunit alpha (COPA), mRNA
CORO1C	2	-12,43					X									coronin, actin binding protein, 1C (CORO1C), mRNA
DHFR	1	-22,32								X						dihydrofolate reductase (DHFR), mRNA
DNMT1	1	-1,84	X		X				X					X		DNA (cytosine-5-)-methyltransferase 1 (DNMT1), mRNA
EGFR	1	-7,16		X	X	X	X	X			X					epidermal growth factor receptor (erythroblastic leukemia viral (v-erb-b) oncogene homolog, avian) (EGFR),
ELAVL1	1	-3,07													X	ELAV (embryonic lethal, abnormal vision, Drosophila)-like 1 (Hu antigen R) (ELAVL1), mRNA
EP300	1	-2,47	X	X		X		X						X		E1A binding protein p300 (EP300), mRNA
EP400	1	-3,43												X		E1A binding protein p400 (EP400), mRNA
ETS1	1	-8,26	X	X		X						X				v-ets erythroblastosis virus E26 oncogene homolog 1 (avian) (ETS1), mRNA
FANCA	1	-16,65			X	X										Fanconi anemia, complementation group A (FANCA), transcript variant 1, mRNA
FZD1	1	-79,02	X													frizzled homolog 1 (Drosophila) (FZD1), mRNA
GMFB	1	-4,33									X					glia maturation factor, beta (GMFB), mRNA
GNRH1	1	-2,90		X				X								gonadotropin-releasing hormone 1 (luteinizing-releasing hormone) (GNRH1), mRNA
H3F3B	1	-4,69												X		H3 histone, family 3B (H3.3B) (H3F3B), mRNA
HLTF	1	-4,51	X										X	X		helicase-like transcription factor (HLTF), transcript variant 1, mRNA
INTS6	1	-2,07													X	integrator complex subunit 6 (INTS6), transcript variant 1, mRNA
IRS1	1	-4,70						X								insulin receptor substrate 1 (IRS1), mRNA
JUB	1	-7,19				X						X				jub, ajuba homolog (Xenopus laevis) (JUB), transcript variant 1, mRNA
KCNIP3	1	-8,61	X	X												Kv channel interacting protein 3, calsenilin (KCNIP3), transcript variant 1, mRNA
LASP1	1	-13,02							X							LIM and SH3 protein 1 (LASP1), mRNA
LIN7C	1	-2,41					X									lin-7 homolog C (C. elegans) (LIN7C), mRNA
LRRCC1	1	-6,04				X										leucine rich repeat and coiled-coil domain containing 1 (LRRCC1), transcript variant 1, mRNA
MECP2	1	-3,52	X													methyl CpG binding protein 2 (Rett syndrome) (MECP2), mRNA
MET	1	-3,40						X			X	X				met proto-oncogene (hepatocyte growth factor receptor) (MET), mRNA
MICB	1	-5,04		X	X											MHC class I polypeptide-related sequence B (MICB), mRNA
MID1	1	-3,92							X				X			midline 1 (Opitz/BBB syndrome) (MID1), transcript variant 3, mRNA
MMD	1	-12,18		X												monocyte to macrophage differentiation-associated (MMD), mRNA
MPDU1	1	-4,86													X	mannose-P-dolichol utilization defect 1 (MPDU1), mRNA
MXD4	1	-6,68	X													MAX dimerization protein 4 (MXD4), mRNA
NBN	1	-4,00			X	X								X		nibrin (NBN), transcript variant 2, mRNA
NCL	1	-1,79													X	nucleolin (NCL), mRNA
NDE1	1	-3,11				X	X		X			X				nudE nuclear distribution gene E homolog 1 (A. nidulans) (NDE1), mRNA
NEDD4	1	-26,59	X		X		X						X			neural precursor cell expressed, developmentally down-regulated 4 (NEDD4), transcript variant 2, mRNA
NEDD9	1	-7,29				X			X							neural precursor cell expressed, developmentally down-regulated 9 (NEDD9), transcript variant 1, mRNA
NRBP2	1	-4,23									X					nuclear receptor binding protein 2 (NRBP2), mRNA
NRP1	1	-14,86							X			X				neuropilin 1 (NRP1), transcript variant 3, mRNA
NT5E	1	-48,13			X					X						5'-nucleotidase, ecto (CD73) (NT5E), mRNA
NUDT21	1	-4,18													X	nudix (nucleoside diphosphate linked moiety X)-type motif 21 (NUDT21), mRNA
NXT2	1	-5,69					X									nuclear transport factor 2-like export factor 2 (NXT2), mRNA
ORC6L	1	-11,48			X											origin recognition complex, subunit 6 like (yeast) (ORC6L), mRNA
OSBPL5	1	-6,32					X									oxysterol binding protein-like 5 (OSBPL5), transcript variant 1, mRNA
PDCD4	1	-7,67	X	X		X		X								programmed cell death 4 (neoplastic transformation inhibitor) (PDCD4), transcript variant 2, mRNA
PIK3C2A	2	-2,51									X					phosphoinositide-3-kinase, class 2, alpha polypeptide (PIK3C2A), mRNA
PPM1F	1	-5,82		X							X					protein phosphatase 1F (PP2C domain containing) (PPM1F), mRNA
PTPLAD1	1	-7,08			X			X			X					protein tyrosine phosphatase-like A domain containing 1 (PTPLAD1), mRNA
PXDN	1	-9,44			X											peroxidasin homolog (Drosophila) (PXDN), mRNA
RAD18	1	-5,18			X								X			RAD18 homolog (S. cerevisiae) (RAD18), mRNA
RAD54B	1	-8,93			X	X										RAD54 homolog B (S. cerevisiae) (RAD54B), mRNA
RFX5	1	-7,36	X													regulatory factor X, 5 (influences HLA class II expression) (RFX5), transcript variant 1, mRNA
RNF138	1	-2,69											X			ring finger protein 138 (RNF138), transcript variant 1, mRNA
RP2	1	-2,29								X						retinitis pigmentosa 2 (X-linked recessive) (RP2), mRNA
RRM2	1	-206,34			X					X						ribonucleotide reductase M2 polypeptide (RRM2), mRNA

RYBP	2	-5,09	X	X												RING1 and YY1 binding protein (RYBP), mRNA
SENP1	1	-6,09											X			SUMO1/sentrin specific peptidase 1 (SENP1), mRNA
SERP1	1	-2,57					X									stress-associated endoplasmic reticulum protein 1 (SERP1), mRNA
SLC23A2	1	-2,33					X									solute carrier family 23 (nucleobase transporters), member 2 (SLC23A2), transcript variant 2, mRNA
SMC4	1	-25,31				X								X		structural maintenance of chromosomes 4 (SMC4), transcript variant 1, mRNA
SNRPD1	1	-4,86													X	small nuclear ribonucleoprotein D1 polypeptide 16kDa (SNRPD1), mRNA
SUZ12	1	-3,31	X											X		suppressor of zeste 12 homolog (Drosophila) (SUZ12), mRNA
TBL1X	1	-6,51	X										X			transducin (beta)-like 1X-linked (TBL1X), mRNA
TFE3	1	-3,35	X													transcription factor binding to IGHM enhancer 3 (TFE3), mRNA
TGFB1I1	1	-2,32											X			transforming growth factor beta 1 induced transcript 1 (TGFB1I1), transcript variant 1, mRNA
THBS1	1	-73,21	X	X	X	X	X	X	X		X	X				thrombospondin 1 (THBS1), mRNA
THG1L	1	-3,71													X	tRNA-histidine guanylyltransferase 1-like (S. cerevisiae) (THG1L), mRNA
TMEM123	1	-9,93		X												transmembrane protein 123 (TMEM123), mRNA
TPM4	1	-4,84										X				tropomyosin 4 (TPM4), mRNA
TRIM4	1	-5,16													X	tripartite motif-containing 4 (TRIM4), transcript variant alpha, mRNA
TRIO	1	-1,98		X							X					triple functional domain (PTPRF interacting) (TRIO), mRNA
TUBB	1	-9,52		X		X			X			X				tubulin, beta (TUBB), mRNA
TWSG1	1	-3,49													X	twisted gastrulation homolog 1 (Drosophila) (TWSG1), mRNA
TYMS	1	-159,68			X					X						thymidylate synthetase (TYMS), mRNA
VPS4B	1	-6,23				X	X	X		X						vacuolar protein sorting 4 homolog B (S. cerevisiae) (VPS4B), mRNA
ZNF462	1	-3,07	X													zinc finger protein 462 (ZNF462), mRNA

miR221	Functional classes (upregulated):												
		p-value	*1,69E-15*	*9,72E-17*	*6,35E-16*	*4,38E-15*	*1,06E-12*	*2,20E-13*	*1,97E-12*	*2,43E-10*		
Target gene name	miRNA sites	Fold change	transcription regulation	ubiquitination/proteolysis	apoptosis	insulin receptor signaling	transport/localization	reg, of kinase activity	cellular respiration	transcription, pos, reg	Other	Gene description
Total: 43			*14*	*13*	*11*	*9*	*9*	*9*	*8*	*7*	*1*	
ACTR3B	1	5,92									X	ARP3 actin-related protein 3 homolog B (yeast) (ACTR3B), transcript variant 2, mRNA
AKAP13	1	10,78			X							A kinase (PRKA) anchor protein 13 (AKAP13), transcript variant 1, mRNA
AMFR	1	3,84		X		X						autocrine motility factor receptor (AMFR), mRNA
BCL2	2	70,63		X	X	X	X	X		X		B-cell CLL/lymphoma 2 (BCL2), nuclear gene encoding mitochondrial protein, transcript variant alpha, mRN
BECN1	1	2,49			X							beclin 1 (coiled-coil, myosin-like BCL2 interacting protein) (BECN1), mRNA
CBX7	1	3,53	X									chromobox homolog 7 (CBX7), mRNA
CDKN2B	1	17,85				X		X				cyclin-dependent kinase inhibitor 2B (p15, inhibits CDK4) (CDKN2B), transcript variant 1, mRNA
CEBPG	1	8,67	X		X			X		X		CCAAT/enhancer binding protein (C/EBP), gamma (CEBPG), mRNA
CHURC1	2	2,77	X							X		churchill domain containing 1 (CHURC1), mRNA
CPEB2	1	9,94		X								cytoplasmic polyadenylation element binding protein 2 (CPEB2), transcript variant B, mRNA
CYCS	3	17,05		X	X			X	X			cytochrome c, somatic (CYCS), nuclear gene encoding mitochondrial protein, mRNA
DHTKD1	1	2,61							X			dehydrogenase E1 and transketolase domain containing 1 (DHTKD1), mRNA
EPM2A	1	42,49							X			epilepsy, progressive myoclonus type 2A, Lafora disease (laforin) (EPM2A), transcript variant 2, mRNA
FAM125B	1	10,28					X					family with sequence similarity 125, member B (FAM125B), transcript variant 2, mRNA
GAN	1	2,85		X	X							giant axonal neuropathy (gigaxonin) (GAN), mRNA
HIF1AN	1	4,60	X						X			hypoxia-inducible factor 1, alpha subunit inhibitor (HIF1AN), mRNA
HMBOX1	1	58,14	X									homeobox containing 1 (HMBOX1), mRNA
IGFBP5	1	6476,31		X								insulin-like growth factor binding protein 5 (IGFBP5), mRNA
KLHL2	1	4,38					X					kelch-like 2, Mayven (Drosophila) (KLHL2), mRNA
KLHL3	1	23,19		X								kelch-like 3 (Drosophila) (KLHL3), mRNA
LNX1	1	8,78		X								ligand of numb-protein X 1 (LNX1), mRNA
MAPK1	1	3,08	X	X	X	X	X	X		X		mitogen-activated protein kinase 1 (MAPK1), transcript variant 1, mRNA
MGEA5	1	3,56		X	X	X	X			X		meningioma expressed antigen 5 (hyaluronidase) (MGEA5), mRNA
NDUFA6	1	3,17							X			NADH dehydrogenase (ubiquinone) 1 alpha subcomplex, 6, 14kDa (NDUFA6), nuclear gene encoding mitoc
NDUFS3	1	4,97			X				X			NADH dehydrogenase (ubiquinone) Fe-S protein 3, 30kDa (NADH-coenzyme Q reductase) (NDUFS3), mRN
NUPL1	1	4,12					X					nucleoporin like 1 (NUPL1), transcript variant 1, mRNA
PDGFD	1	11,98		X				X				platelet derived growth factor D (PDGFD), transcript variant 1, mRNA
PTPN11	1	4,61				X		X				protein tyrosine phosphatase, non-receptor type 11 (Noonan syndrome 1) (PTPN11), mRNA
RAB23	1	6,25		X			X					RAB23, member RAS oncogene family (RAB23), transcript variant 1, mRNA
SETD7	1	36,80	X									SET domain containing (lysine methyltransferase) 7 (SETD7), mRNA
SLC25A4	1	704,27			X				X			solute carrier family 25 (mitochondrial carrier; adenine nucleotide translocator), member 4 (SLC25A4), nucle
SNX9	1	3,31					X					sorting nexin 9 (SNX9), mRNA
SOCS4	2	3,59		X								suppressor of cytokine signaling 4 (SOCS4), transcript variant 1, mRNA
STAT3	2	7,94	X			X		X		X		signal transducer and activator of transcription 3 (acute-phase response factor) (STAT3), transcript variant 3,
STAT5B	2	10,87	X		X	X		X		X		signal transducer and activator of transcription 5B (STAT5B), mRNA
TCF20	1	2,20	X									transcription factor 20 (AR1) (TCF20), transcript variant 1, mRNA
TFRC	1	21,46				X						transferrin receptor (p90, CD71) (TFRC), mRNA
VPS13D	1	2,08					X					vacuolar protein sorting 13 homolog D (S. cerevisiae) (VPS13D), transcript variant 1, mRNA
ZADH2	1	2,41							X			zinc binding alcohol dehydrogenase, domain containing 2 (ZADH2), mRNA
ZBTB47	2	29,55	X									zinc finger and BTB domain containing 47 (ZBTB47), mRNA
ZFP28	1	2,02	X									zinc finger protein 28 homolog (mouse) (ZFP28), mRNA
ZNF302	1	76,69	X									zinc finger protein 302 (ZNF302), transcript variant 1, mRNA
ZNF780B	1	6,46	X									zinc finger protein 780B (ZNF780B), mRNA

miR222	Functional classes (upregulated):										
		p-value	*1,33E-26*	*7,74E-22*	*1,08E-18*	*5,15E-19*	*1,07E-16*	*2,31E-15*	*1,02E-11*		
Target gene name	miRNA sites	Fold change	ubiquitination/proteolysis	transcription regulation	apoptosis	reg, of kinase activity	transport/localization	cellular respiration	transcription, pos, reg	Other	Gene description
Total: 72			*21*	*20*	*13*	*13*	*12*	*10*	*8*	*9*	
AKAP13	2	10,78			X						A kinase (PRKA) anchor protein 13 (AKAP13), transcript variant 1, mRNA
ASB8	1	4,18	X								ankyrin repeat and SOCS box-containing 8 (ASB8), mRNA
BACH2	1	18,94		X							BTB and CNC homology 1, basic leucine zipper transcription factor 2 (BACH2), mRNA
BBC3	1	13,63			X	X					BCL2 binding component 3 (BBC3), mRNA
BDP1	1	2,63		X							B double prime 1, subunit of RNA polymerase III transcription initiation factor IIIB (BDP1), mRNA
CEBPG	1	8,67		X	X	X			X		CCAAT/enhancer binding protein (C/EBP), gamma (CEBPG), mRNA
CPEB2	1	9,94	X								cytoplasmic polyadenylation element binding protein 2 (CPEB2), transcript variant B, mRNA
CYCS	1	17,05	X		X	X		X			cytochrome c, somatic (CYCS), nuclear gene encoding mitochondrial protein, mRNA
DGKI	1	18,83				X					diacylglycerol kinase, iota (DGKI), mRNA
E2F5	1	6,67		X							E2F transcription factor 5, p130-binding (E2F5), mRNA
EIF5	1	2,56	X								eukaryotic translation initiation factor 5 (EIF5), transcript variant 1, mRNA
EPB41L5	1	20,30								X	erythrocyte membrane protein band 4.1 like 5 (EPB41L5), mRNA
EPM2A	1	42,49						X			epilepsy, progressive myoclonus type 2A, Lafora disease (laforin) (EPM2A), transcript variant 2, mRNA
FAM125B	2	10,28					X				family with sequence similarity 125, member B (FAM125B), transcript variant 2, mRNA
FBXO27	1	9,71	X								F-box protein 27 (FBXO27), mRNA
GAN	1	2,85	X		X						giant axonal neuropathy (gigaxonin) (GAN), mRNA
GGA3	1	1,99					X				golgi associated, gamma adaptin ear containing, ARF binding protein 3 (GGA3), transcript variant long, mRN
HIF1AN	1	4,60		X				X			hypoxia-inducible factor 1, alpha subunit inhibitor (HIF1AN), mRNA
HMBOX1	1	58,14		X							homeobox containing 1 (HMBOX1), mRNA
IGFBP5	1	6476,31	X								insulin-like growth factor binding protein 5 (IGFBP5), mRNA
KLHL2	1	4,38					X				kelch-like 2, Mayven (Drosophila) (KLHL2), mRNA
KLHL3	2	23,19	X								kelch-like 3 (Drosophila) (KLHL3), mRNA
L2HGDH	1	3,17						X			L-2-hydroxyglutarate dehydrogenase (L2HGDH), nuclear gene encoding mitochondrial protein, mRNA
LNX1	1	8,78	X								ligand of numb-protein X 1 (LNX1), mRNA
MAFF	1	3,53		X							v-maf musculoaponeurotic fibrosarcoma oncogene homolog F (avian) (MAFF), transcript variant 1, mRNA
MAP1A	1	4,76								X	microtubule-associated protein 1A (MAP1A), mRNA
MAPK1	1	3,08	X	X	X	X	X		X		mitogen-activated protein kinase 1 (MAPK1), transcript variant 1, mRNA
MEF2C	1	23,94		X	X				X		MADS box transcription enhancer factor 2, polypeptide C (myocyte enhancer factor 2C) (MEF2C), mRNA
MYOM2	1	9455,53								X	myomesin (M-protein) 2, 165kDa (MYOM2), mRNA
NDUFA6	1	3,17						X			NADH dehydrogenase (ubiquinone) 1 alpha subcomplex, 6, 14kDa (NDUFA6), nuclear gene encoding mitoc
NDUFS3	1	4,97			X			X			NADH dehydrogenase (ubiquinone) Fe-S protein 3, 30kDa (NADH-coenzyme Q reductase) (NDUFS3), mRN
NMNAT2	1	26,05								X	nicotinamide nucleotide adenylyltransferase 2 (NMNAT2), transcript variant 1, mRNA
PANK3	1	4,96								X	pantothenate kinase 3 (PANK3), mRNA
PCGF3	2	3,34		X							polycomb group ring finger 3 (PCGF3), mRNA
PDGFD	1	11,98	X			X					platelet derived growth factor D (PDGFD), transcript variant 1, mRNA
PLAGL1	2	6,61		X	X				X		pleiomorphic adenoma gene-like 1 (PLAGL1), transcript variant 2, mRNA
PPM1E	2	313,73									protein phosphatase 1E (PP2C domain containing) (PPM1E), mRNA
PRICKLE2	3	10,19								X	prickle homolog 2 (Drosophila) (PRICKLE2), mRNA
PSMB5	1	6,02	X			X			X		proteasome (prosome, macropain) subunit, beta type, 5 (PSMB5), mRNA
PTPN11	2	4,61				X					protein tyrosine phosphatase, non-receptor type 11 (Noonan syndrome 1) (PTPN11), mRNA
RAB18	1	2,86		X			X				RAB18, member RAS oncogene family (RAB18), mRNA
RAB23	1	6,25	X				X				RAB23, member RAS oncogene family (RAB23), transcript variant 1, mRNA
RAB6B	1	8,48					X				RAB6B, member RAS oncogene family (RAB6B), mRNA
RAB9B	1	264,01					X				RAB9B, member RAS oncogene family (RAB9B), mRNA
RNF41	1	86,27	X								ring finger protein 41 (RNF41), transcript variant 2, mRNA
RNPEPL1	1	4,42	X								arginyl aminopeptidase (aminopeptidase B)-like 1 (RNPEPL1), mRNA
RRM2B	1	8,04			X			X			ribonucleotide reductase M2 B (TP53 inducible) (RRM2B), mRNA
SETD7	1	36,80		X							SET domain containing (lysine methyltransferase) 7 (SETD7), mRNA
SGCG	1	12133,22								X	sarcoglycan, gamma (35kDa dystrophin-associated glycoprotein) (SGCG), mRNA
SIAH1	1	2,13	X		X						seven in absentia homolog 1 (Drosophila) (SIAH1), transcript variant 1, mRNA
SLC11A2	1	3,30			X	X					solute carrier family 11 (proton-coupled divalent metal ion transporters), member 2 (SLC11A2), mRNA
SLC25A12	1	23,14					X	X			solute carrier family 25 (mitochondrial carrier, Aralar), member 12 (SLC25A12), mRNA
SLC25A4	1	704,27			X			X			solute carrier family 25 (mitochondrial carrier; adenine nucleotide translocator), member 4 (SLC25A4), nucle
SNX9	1	3,31					X				sorting nexin 9 (SNX9), mRNA
SOCS4	1	3,59	X								suppressor of cytokine signaling 4 (SOCS4), transcript variant 1, mRNA
SOCS5	1	2,00	X								suppressor of cytokine signaling 5 (SOCS5), transcript variant 2, mRNA
SPRED2	1	3,86				X					sprouty-related, EVH1 domain containing 2 (SPRED2), mRNA
STAT3	1	7,94		X		X			X		signal transducer and activator of transcription 3 (acute-phase response factor) (STAT3), transcript variant 3,
TBL1XR1	1	2,13	X	X					X		transducin (beta)-like 1X-linked receptor 1 (TBL1XR1), mRNA
TMOD1	1	6927,37								X	tropomodulin 1 (TMOD1), mRNA
TOLLIP	1	2,85									toll interacting protein (TOLLIP), mRNA
TRIM72	1	6,46								X	tripartite motif-containing 72 (TRIM72), mRNA
UBE2D1	1	7,44	X			X			X		ubiquitin-conjugating enzyme E2D 1 (UBC4/5 homolog, yeast) (UBE2D1), mRNA
VGLL4	1	8,67		X							vestigial like 4 (Drosophila) (VGLL4), mRNA
VPS24	2	5,04					X				vacuolar protein sorting 24 homolog (S. cerevisiae) (VPS24), transcript variant 1, mRNA
YWHAG	1	4,09				X	X				tyrosine 3-monooxygenase/tryptophan 5-monooxygenase activation protein, gamma polypeptide (YWHAG),

ZADH2	1	2,41						X			zinc binding alcohol dehydrogenase, domain containing 2 (ZADH2), mRNA
ZBTB47	2	29,55		X							zinc finger and BTB domain containing 47 (ZBTB47), mRNA
ZFP28	1	2,02		X							zinc finger protein 28 homolog (mouse) (ZFP28), mRNA
ZNF652	1	3,77		X							zinc finger protein 652 (ZNF652), mRNA
ZNF780B	1	6,46		X							zinc finger protein 780B (ZNF780B), mRNA
ZRANB1	1	7,47	X								zinc finger, RAN-binding domain containing 1 (ZRANB1), mRNA

**miR550**	Functional classes (upregulated):												
		p-value	*3,39E-12*	*4,62E-11*	*2,03E-07*	*6,33E-08*	*6,19E-08*	*2,39E-07*	*1,43E-05*	*6,73E-05*	*1,01E-05*		
Target gene name	miRNA sites	Fold change	transcription, pos, reg	ubiquitination/proteolysis	transcription regulation	phosphate metabolism	apoptosis	insulin receptor signaling	muscle development	transport/localization	cell morphogenesis	Other	Gene description
Total: 24			*8*	*8*	*6*	*5*	*5*	*4*	*3*	*3*	*3*	*4*	
AFF1	1	2,23	X		X								AF4/FMR2 family, member 1 (AFF1), mRNA
CASQ2	1	44556,70							X				calsequestrin 2 (cardiac muscle) (CASQ2), mRNA
DLD	1	2,63		X		X							dihydrolipoamide dehydrogenase (DLD), mRNA
DYRK2	2	6,71	X			X	X						dual-specificity tyrosine-(Y)-phosphorylation regulated kinase 2 (DYRK2), transcript variant 2, mRNA
EIF5	1	2,56		X									eukaryotic translation initiation factor 5 (EIF5), transcript variant 1, mRNA
ERBB3	1	4,22				X	X	X	X		X		v-erb-b2 erythroblastic leukemia viral oncogene homolog 3 (avian) (ERBB3), transcript variant 1, mRNA
FBXO27	1	9,71		X									F-box protein 27 (FBXO27), mRNA
FOSL2	1	2,75	X		X		X						FOS-like antigen 2 (FOSL2), mRNA
H1F0	1	35,31										X	H1 histone family, member 0 (H1F0), mRNA
LPIN1	1	10,79						X			X		lipin 1 (LPIN1), mRNA
MAPK1	2	3,08	X	X	X	X	X	X		X			mitogen-activated protein kinase 1 (MAPK1), transcript variant 1, mRNA
MGEA5	1	3,56	X	X			X	X		X			meningioma expressed antigen 5 (hyaluronidase) (MGEA5), mRNA
MSRB3	1	3,49										X	methionine sulfoxide reductase B3 (MSRB3), transcript variant 2, mRNA
PANK3	1	4,96										X	pantothenate kinase 3 (PANK3), mRNA
PPM1A	1	10,68	X		X	X							protein phosphatase 1A (formerly 2C), magnesium-dependent, alpha isoform (PPM1A), transcript variant 1, r
PRICKLE2	1	10,19									X		prickle homolog 2 (Drosophila) (PRICKLE2), mRNA
RCOR2	1	1,99			X								REST corepressor 2 (RCOR2), mRNA
SAMD4A	1	25,92	X	X									sterile alpha motif domain containing 4A (SAMD4A), mRNA
SOCS5	1	2,00		X									suppressor of cytokine signaling 5 (SOCS5), transcript variant 2, mRNA
SOX11	1	37,36	X		X								SRY (sex determining region Y)-box 11 (SOX11), mRNA
TTL	1	18,69										X	tubulin tyrosine ligase (TTL), mRNA
XPOT	1	3,93								X			exportin, tRNA (nuclear export receptor for tRNAs) (XPOT), mRNA
ZFAND5	1	6,86							X				zinc finger, AN1-type domain 5 (ZFAND5), mRNA
ZRANB1	1	7,47		X									zinc finger, RAN-binding domain containing 1 (ZRANB1), mRNA
