# Supplementary material for: Simultaneous miRNA and mRNA transcriptome profiling of human myoblasts reveals a novel set of myogenic differentiation-associated miRNAs and their target genes
Source: BMC Genomics. 2013 Apr 18;14:265. doi: 10.1186/1471-2164-14-265 (PMC3639941; doi:10.1186/1471-2164-14-265)
Supplement: Additional file 10: Table S7 — microRNAs that were previously shown to be differentially expressed during myogenesis but could not be confirmed in the present study. [file 1471-2164-14-265-S10.docx]

**Table S7**. microRNAs that were previously shown to be differentially expressed during myogenesis but could not be confirmed in the present study. From the [1] microRNAs we deduced p-value of student's t-test from available expression data and selected microRNAs with p-value <0.05.

| microRNA | human | mouse | pig | carp |
| --- | --- | --- | --- | --- |
| let-7c |  |  | ↓ muscle development [2] |  |
| let-7e |  | ↑ pMyo diff [3] |  |  |
| let-7i |  |  | ↓ muscle development [2] |  |
| let-7f |  | ↑ pMyo diff [3] |  |  |
| let-7g |  | ↑C2C12 diff [3] |  |  |
| miR-7 | ↓ pMyo diff. [1] |  |  |  |
| miR-10b | ↑pMyo diff. [4] |  |  |  |
| miR-15b | ↓ pMyo diff. [1] | ↓ C2C12 diff [5] [3] ↓ pMyo diff [3] |  |  |
| miR-17 | ↑ pMyo diff. [1] ↑pMyo diff. [4] | ↓ C2C12 diff [3] |  |  |
| miR-18a |  | ↓ C2C12 diff [5] [3] ↓ pMyo diff [3] |  |  |
| miR-19a | ↑pMyo diff. [4] ↓ pMyo diff. [1] | ↓ C2C12 diff [3] ↓ pMyo diff [3] |  |  |
| miR-19b | ↑pMyo diff. [4] | ↓ pMyo diff [3] |  |  |
| miR-20a/b | ↓ pMyo diff. [1] | ↓ C2C12 diff [5] [3] ↓ pMyo diff [3] |  |  |
| miR-23a | ↑ pMyo diff. [1] |  |  | enriched in sk. muscle [6] |
| miR-25 |  | ↓ C2C12 diff [3] ↓ pMyo diff [3] |  |  |
| miR-29b/c | ↑pMyo diff. [7] | ↑C2C12 diff [7] [3] |  |  |
| miR-30b |  | ↑ pMyo diff [3] | ↑ muscle development [2] |  |
| miR-30c |  |  |  |  |
| miR-34a |  | ↑C2C12 diff [3] ↑ pMyo diff [3] |  |  |
| miR-34c |  | ↑C2C12 diff [3] |  |  |
| miR-92a |  | ↓ C2C12 diff [3] ↓ pMyo diff [3] |  |  |
| miR-93 | ↓ pMyo diff. [1] | ↓ C2C12 diff [3] ↓ pMyo diff [3] |  |  |
| miR-100 |  | ↑C2C12 diff [3] ↑ pMyo diff [3] |  |  |
| miR-106a | ↑pMyo diff. [4] | ↓ C2C12 diff [3] ↓ pMyo diff [3] |  |  |
| miR-106b | ↓ pMyo diff. [1] | ↓ C2C12 diff [3] ↓ pMyo diff [3] |  |  |
| miR-122 |  |  | ↓ muscle development [2] |  |
| miR-124a | ↑pMyo diff. [4] |  |  |  |
| miR-125b |  | ↓ C2C12 diff [8] ↓ C2C12 diff [5] |  |  |
| miR-130a |  |  | ↓ muscle development [2] |  |
| miR-130b | ↓ pMyo diff. [1] |  |  |  |
| miR-132 |  | ↑ C2C12 diff [5] ↓ pMyo diff [3] |  |  |
| miR-137 | ↓ pMyo diff. [1] |  |  |  |
| miR-138 |  | ↑ pMyo diff [3]↑ pMyo diff [3] |  |  |
| miR-139 | ↑ pMyo diff. [1] | ↑ C2C12 diff [3] ↑ pMyo diff [3] | ↓ muscle development [2] |  |
| miR-141 | ↑pMyo diff. [4] |  |  |  |
| miR-143 | enriched in sk. muscle and the heart [9] ↑ pMyo diff. [1] | enriched in sk. muscle and the heart [9] ↑ C2C12 diff [5] [3] ↑ pMyo diff [3] |  |  |
| miR-145 | ↑ pMyo diff. [1] | ↑ C2C12 diff [5] [3] ↑ pMyo diff [3] |  |  |
| miR-146b |  | ↑ C2C12 diff [5] |  |  |
| miR-148a |  |  | ↓ muscle development [2] |  |
| miR-149 |  | ↑ pMyo diff [3] |  |  |
| miR-150 |  | ↓ C2C12 diff [5] |  |  |
| miR-151a/b |  |  | ↑ muscle development [2] |  |
| miR-181a |  | ↑ C2C12 diff [3] ↑ pMyo diff [3] |  |  |
| miR-181c |  | ↑ C2C12 diff [3] ↑ pMyo diff [3] |  |  |
| miR-181d |  | ↑ C2C12 diff [3] ↑ pMyo diff [3] |  |  |
| miR-182 | ↓ pMyo diff. [1] | ↓ pMyo diff [3] |  |  |
| miR-183 |  | ↓ pMyo diff [3] |  |  |
| miR-184 | ↑ pMyo diff. [1] |  |  |  |
| miR-186 |  | ↓ C2C12 diff [3] |  |  |
| miR-188 |  | ↑ pMyo diff [3] |  |  |
| miR-191 |  | ↑ pMyo diff [3] |  |  |
| miR-193b |  | ↑ pMyo diff [3] |  |  |
| miR-194 |  | ↑ pMyo diff [3] |  |  |
| miR-195 |  | ↓ pMyo diff [3] |  |  |
| miR-196b |  | ↓ C2C12 diff [3] ↓ pMyo diff [3] |  |  |
| miR-199 |  | ↑ C2C12 diff [5] | ↓ muscle development [2] |  |
| miR-200b | ↑ pMyo diff. [1] |  |  |  |
| miR-200c | ↑pMyo diff. [4] |  |  |  |
| miR-203 | ↑pMyo diff. [4] |  |  |  |
| miR-208a | enriched in sk. muscle and the heart [9] ↑ pMyo diff. [1] | enriched in sk. muscle and the heart [9] |  |  |
| miR-208b | [this study] | enriched in sk. muscle and the heart [10] |  |  |
| miR-214 | [this study] | enriched in mouse primary myoblasts and tissues [11] ↑ C2C12 diff [5] | ↓ muscle development [2] | ↓ muscle development [6] |
| miR-218 |  | ↑ C2C12 diff [5] ↓ pMyo diff [3] |  |  |
| miR-299 | ↑ pMyo diff. [1] |  |  |  |
| miR-301 | ↑pMyo diff. [4] |  |  |  |
| miR-301b |  | ↓ C2C12 diff [3] |  |  |
| mmu-miR-322  /hsa-miR-424 | [this study] | ↑ C2C12 diff [12] [5] ↑ pMyo diff [3] |  |  |
| miR-323 |  |  | ↓ muscle development [2] |  |
| miR-326 |  | ↓ C2C12 diff [5] |  |  |
| miR-328 |  | ↑ C2C12 diff [3] ↑ pMyo diff [3] |  |  |
| miR-335 |  | ↑ C2C12 diff [5] |  |  |
| miR-342 |  | ↓ C2C12 diff [3] |  |  |
| miR-351 |  | ↑ C2C12 diff [5] [3] ↑ pMyo diff [3] |  |  |
| miR-365 |  | ↑ C2C12 diff [3] ↑ pMyo diff [3] | ↑ muscle development [2] |  |
| miR-378 | ↑ pMyo diff. [1] | ↑ C2C12 diff [5] [3] ↑ pMyo diff [3] |  |  |
| miR-409 |  | ↑ pMyo diff [3] |  |  |
| miR-422a | ↑ pMyo diff. [1] |  |  |  |
| miR-423 |  | ↓ C2C12 diff [3] ↑ pMyo diff [3] |  |  |
| miR-450a |  | ↑ C2C12 diff [5] |  |  |
| miR-466g-c/b |  |  | ↓ muscle development [2] |  |
| miR-486 | ↓ pMyo diff. [1] | ↑ C2C12 diff [3] ↑ pMyo diff [3] |  |  |
|  |  |  |  |  |
| miR-491 | ↑ pMyo diff. [1] |  |  |  |
| miR-494 |  | ↑ pMyo diff [3] |  |  |
| miR-497 |  | ↓ C2C12 diff [3] ↓ pMyo diff [3] |  |  |
| miR-499 |  | enriched in sk. muscle and the heart [10] ↑ C2C12 diff [5] ↑ pMyo diff [3] |  |  |
| miR-504 | ↑ pMyo diff. [1] |  |  |  |
| miR-545 | ↑ pMyo diff. [1] |  |  |  |
| miR-652 |  | ↓ pMyo diff [3] |  |  |
| miR-654 | ↑ pMyo diff. [1] |  |  |  |
| miR-576 | ↓ pMyo diff. [1] |  |  |  |
| miR-665d |  |  | ↓ muscle development [2] |  |
| miuR-667 |  | ↑ pMyo diff [3] |  |  |
| miR-675-3p |  | ↑ C2C12 diff [3] ↑ pMyo diff [3] |  |  |
| miR-677 |  | ↓ C2C12 diff [5] |  |  |
| miR-682 | [this study] | enriched in murine myogenic progenitors [13] |  |  |
| miR-720a/b |  |  | ↑ muscle development [2] |  |
| miR-744 |  | ↑ C2C12 diff [3] ↑ pMyo diff [3] |  |  |
| miR-762 |  | ↓ C2C12 diff [5] |  |  |
| miR-805 |  | ↓ C2C12 diff [5] |  |  |
| miR-923 |  |  | ↓ muscle development [2] |  |
| miR-1280a/b |  |  | ↑ muscle development [2] |  |
| miRPlus_17653 |  | ↑ C2C12 diff [5] |  |  |
| miRPlus_27561 |  | ↓ C2C12 diff [5] |  |  |
| miRPlus_17890 |  | ↓ C2C12 diff [5] |  |  |
| miRPlus_17833 |  | ↓ C2C12 diff [5] |  |  |
